# A Meta-Analysis of Job Insecurity and Employee Performance: Testing Temporal Aspects, Rating Source, Welfare Regime, and Union Density as Moderators

**DOI:** 10.3390/ijerph16142536

**Published:** 2019-07-16

**Authors:** Magnus Sverke, Lena Låstad, Johnny Hellgren, Anne Richter, Katharina Näswall

**Affiliations:** 1 Department of Psychology, Stockholm University, 10691 Stockholm, Sweden; 2 Department of Psychology, University of Gothenburg, Box 100, 40530 Gothenburg, Sweden; 3 Department of Learning, Informatics, Management and Ethics (LIME), Karolinska Institutet, 17177 Stockholm, Sweden; 4 School of Psychology, Speech and Hearing, University of Canterbury, Private Bag 4800, 8140 Christchurch, New Zealand

**Keywords:** job insecurity, job performance, contextual performance, counterproductive work behavior, creativity, safety compliance, supervisor ratings, longitudinal, union density, welfare regime

## Abstract

Previous research has shown that job insecurity is linked to a range of performance outcomes, but the number of studies exploring this relationship is still limited and the results are somewhat mixed. The first aim of this study was to meta-analytically investigate how job insecurity is related to task performance, contextual performance, counterproductive work behavior, creativity, and safety compliance. The second aim was to test two method-related factors (cross-sectional vs. longitudinal associations and self- vs. supervisor-ratings of performance) and two macro-level indicators of social protection (social welfare regime and union density) as moderators of these associations. The results show that job insecurity was generally associated with impaired employee performance. These findings were generally similar both cross-sectionally and longitudinally and irrespective of rater. Overall, the associations between job insecurity and negative performance outcomes were weaker in welfare regimes characterized by strong social protection, whereas the results concerning union density produced mixed results. A majority of the findings confirmed the negative associations between job insecurity and types of employee performance, but future research is needed to elaborate on the effects of temporal aspects, differences between ratings sources, and further indicators of social protection in different cultural settings in the context of job insecurity.

## 1. Introduction

Many employees worry about potential job loss. This is apparent from both population-based empirical studies [1,2] and a number of recently published literature reviews and meta-analyses [3,4,5,6]. There are many drivers of this development, including technological advancements, increased globalized competition, and a general ambition within organizations to increase effectiveness and save costs [7,8]. While a large number of studies have investigated the detrimental effects of job insecurity on work-related attitudes and health and wellbeing outcomes, behavioral outcomes have been studied to a somewhat lesser extent. Especially calling for more attention is employee performance at work, as it is a central behavior that has the potential to affect organizational functioning and survival in competitive markets. While previous meta-analytical results indicate that the overall association between job insecurity and employee performance is negative [4,9,10], positive associations have also been reported [11,12,13]. Mirroring this duality, the link between job insecurity and employee performance has generally been theorized in two contrasting ways in previous studies. Whereas the bulk of theoretical approaches suggests that job insecurity, like any stressor, would result in negative consequences, such as impaired performance through employee withdrawal [14,15], others maintain that job insecurity may challenge employees to demonstrate their value to the organization by performing better and engaging less in counterproductive work behaviors [16,17]. This inconsistency could be due to the way in which employee performance was operationalized in these previous meta-analyses. In comparison, the present study utilizes a wider range of performance outcomes, which resulted in more primary studies being included, and in turn, a more nuanced understanding of the investigated relationships. More specifically, by meta-analytically investigating how job insecurity relates to five different types of employee performance—task performance, contextual performance, creativity, counterproductive work behaviors, and safety performance—we were able to make progress in uncovering what underlies the sometimes inconsistent findings, thus contributing to knowledge on the workings and impact of job insecurity.

It is not only the theorizing on the link between job insecurity and employee performance that has taken differing directions, but also the discussion surrounding the most appropriate type of study design for further examination of this link. The second contribution of this study is the testing of two method-related moderators. One of these concerns temporal aspects, and in particular, whether the strength of the associations varies over time, as discussed in previous reviews [3,18] and meta-analyses [19] on job insecurity. By testing whether study design, that is, cross-sectional or longitudinal, moderates the strength of the associations, the present study contributes to a better understanding of the job insecurity–performance link. The second focus of method-related moderator testing was on the types of performance ratings used. It has been suggested that self-ratings and supervisor-ratings of job performance differ systematically, in the sense that employees may be motivated to rate their own performance more favorably than their supervisors [20]. This may be particularly true in situations where the job is at risk, resulting in a weaker negative, or even positive correlation between job insecurity and self-rated performance compared to supervisor-rated performance. It can also be expected that job insecurity and self-ratings of performance are more strongly negatively correlated than job insecurity and supervisor-ratings of performance due to mono-method bias [21]. By testing the moderating effect of self-ratings, as compared to supervisor-ratings, this study may have important implications for future research on the link between job insecurity and employee performance.

Despite the importance of performance-related outcomes, it is still unclear under which conditions job insecurity is associated with impaired performance. It has been suggested that the nature and strength of this relationship may differ depending on macro-level factors related to the social safety net of workers [6,22]. The third contribution of the present meta-analysis is the testing of two macro-level indicators of social protection (social welfare regime and union density) as moderators of the association between job insecurity and employee performance. Regarding the type of welfare regime, it has been argued that, in countries that have an extensive welfare system, employees are better protected if they become unemployed or if their job is at risk [23]. This suggests that the consequences of job insecurity could be expected to differ between national contexts [24]. A further aspect related to social protection is the strength of labor unions. Differences in union density among countries may produce differing degrees of protection against job loss or the risk of job loss among their employed populations [25,26]. It can be expected that the relationship between job insecurity and performance is weaker in countries with stronger social protection systems. 

### 1.1. Nature of Job Insecurity

The most-quoted definition of job insecurity was presented by Greenhalgh and Rosenblatt [27], who defined job insecurity as “the perceived powerlessness to maintain desired continuity in a threatened job situation” (p. 438). This definition has guided the scholarly understanding of job insecurity, and has served as a point of departure for the many subsequent expansions of this definition that have been put forward, where a recent overview of definitions has been provided by Shoss [6]. Most definitions of job insecurity include some, or all, of the following characteristics: (a) It is a subjective perception of a threat to the job [28]; (b) the changes to the job implied by the threat are unwanted and involuntary [29,30]; and (c) it entails a feeling of concern or apprehension about losing one’s job in its current form [31,32]. Some definitions treat job insecurity as multi-dimensional, differentiating between qualitative (threats to valued job features) and quantitative (threats to the continuation of employment) job insecurity [6,33]. Another distinction often made is between cognitive and affective job insecurity [31,34], where the former refers to the perception of a threat to the job (or job features) and the latter concerns the emotional reaction to such a threat, e.g., worry and anxiety [4]. In the present study, we treat job insecurity as a global construct comprising a perceived, unwelcome threat to the current job, see also Probst [35] for a similar conceptualization.

Job insecurity has consistently been described as something problematic and unpleasant for individuals to experience, as it has been found to have negative consequences both cross-sectionally [4,9,19] and longitudinally [3]. These negative consequences can be explained by different, yet somewhat interrelated, theoretical frameworks. One of the more common theoretical explanations for why job insecurity has negative consequences views job insecurity as a stressor, in that it presents the individual with a demand that may exceed the individual’s resources to handle it. The transactional stress theory [36] describes the process of appraisal, where individuals determine what a situation or a stimulus entails for them, and if it is perceived as threatening, what can be done about the threat. In the case of job insecurity, the threat presented is vague and the source may be uncertain, and it may be unclear what can and should be done to counteract the threat; such an appraisal is likely to result in stress reactions [19,37]. The threat associated with job insecurity is not necessarily just about the job and potentially becoming unemployed. For example, it has been suggested [38] that job insecurity constitutes a threat against latent benefits of work, such as collective purpose, status, social contact, time structure, and engagement in activity, in addition to the threat to a steady income [39]. Furthermore, job insecurity has also been described as a threat to an employee’s identity as an employed person [40]. Job insecurity may also threaten an employee’s ability to retain and gain resources, as described by the Conservation of Resources (COR) theory [41], in that the job represents an important resource under threat for those experiencing job insecurity [6].

The negative consequences of job insecurity have also been explained in relation to a breach of the psychological contract between employee and employer. The psychological contract encompasses unspoken expectations of reciprocal obligations between employee and employer [42]: For example, a shared expectation is that when an employee does a good job and performs well, the employer will ensure that the employee keeps their job [43]. Job insecurity, as it constitutes a change in the perceived contract from secure to insecure, may therefore constitute a perceived breach of the psychological contract [44]. Such a breach can be expected to have negative consequences, as psychological contract breach has been associated with strong negative emotional reactions [45].

Consistent with the mechanisms described above, job insecurity has consistently been associated with stress-related outcomes such as mental and physical health complaints [5,9,19] as well as outcomes focused on the relationship between employee and organization, such as distrust [46], decreased job satisfaction, and impaired commitment [4,9]. Less consistent across empirical studies, however, are findings on the relationship between job insecurity and employee performance. Next, we define employee performance, and then turn to the empirical evidence regarding the association between job insecurity and performance.

### 1.2. Employee Performance

Employee performance is an umbrella term encompassing various types of job performance, but may also include broader categories of work-related behavior reflecting how the job is being carried out. According to a classic definition, job performance is conceptualized as the expected value to an organization of an individual’s multiple accumulated behaviors over a certain time period, where an individual may engage in various sets of behaviors that impact organizational effectiveness in positive or negative ways [47]. While definitions of job performance exist, a widely used definition is based on Campbell’s [48] model of job performance. Campbell defines job performance as individual behaviors and actions that are relevant for the organization’s abilities to reach its goals. In contemporary research, job performance is usually regarded as a phenomenon consisting of three distinct types, or dimensions, of performance behaviors—task performance, contextual performance, and counterproductive work behavior [49,50]. 

Task performance concerns the carrying out of work responsibilities, assessed as how well an employee performs their central job tasks. It concerns behaviors that relate to the employee’s way of performing specific work tasks that are specified in a job description or communicated to the employee in other ways [51]. Individuals’ proficiency in carrying out their tasks, sometimes called in-role behavior, can concern both the quantity and the quality of the task performance.

Contextual performance refers to behaviors that contribute to the organization’s goal fulfillment through its positive impact on organizational, social, and psychological aspects of the working climate [52]. This type of performance, sometimes called organizational citizenship behavior [53], can roughly be divided into two different categories. One of these includes behaviors that enable the organization to function as smoothly as possible (e.g., helping colleagues when needed and contributing to a good atmosphere and a good work environment in the organization), while the other relates to behaviors intended to promote changes and improvements to the organization’s work processes (e.g., being solution-oriented and participating in various projects). However, these two dimensions are often considered a general expression of citizenship behavior and it has been shown that they can best be measured as a uni-dimensional phenomenon [52]. 

Counterproductive work behaviors represent the negative side of the job performance concept and relate to behaviors that have an inhibitory impact on the organization’s ability to reach its goals [54]. Examples of such deviant and non-compliant job behavior are theft, negligence regarding materials or equipment, knowledge hiding, tardiness, unreasonable absence, bullying, and other forms of harassment. 

While contextual performance does incorporate many pro-organizational behaviors, certain types of pro-organizational behaviors are sometimes defined as separate dimensions of the job performance concept, such as creativity [55]. For example, it has been argued that organizations are dependent on employees’ creativity and innovation in order to be competitive and successful [56]. This has led research to argue for an expansion of the performance concept to also include creativity and innovation as dimensions of work performance [55,57]. Following this reasoning, a recent meta-analysis [55] investigated how creative and innovative performance (CIP) relates to task performance, organizational citizenship behavior (OCB, i.e., contextual performance), and counterproductive work behavior. The results revealed that the correlations between CIP and both task performance and OCB were both weaker than the correlation between task performance and OCB, and the authors suggest that an empirical distinction be made between CIP and the other performance dimensions, especially considering its particular potential to generally affect organizational performance [55,56]. Therefore, creativity is treated as a separate dimension of employee performance in the present analysis and refers to innovative thinking and idea generation as well as to the implementation of new ideas into organizational routines—behaviors that are essential to organizational effectiveness in competitive markets [56], and hence vital for organizational performance in general [55].

Another central aspect of employee performance concerns safety performance [58]. While safety-related behaviors are sometimes included in the broader construct of contextual performance, safety behaviors and safety compliance are typically considered an autonomous dimension of employee performance. Safety performance refers to work behaviors that are conducted in accordance with security and safety regulations within the organization. However, non-compliant safety behavior can also be seen as counterproductive work behaviors since violation of safety-related rules and procedures jeopardizes individual as well as organizational health and safety [59]. In the present meta-analysis, we have separated safety performance from other types of employee performance, enabling a more detailed investigation of how job insecurity relates to different types of performance-related behaviors that may be negative for the organization as well as the employee.

### 1.3. Job Insecurity and Employee Performance

The theoretical approaches used to explain why job insecurity may lead to various types of consequences—such as stress theory [36], psychological contract theory [42], and identity theory [40]—have also been used with respect to employee performance. By applying the stress theory, for instance, experiencing stress and uncertainty over the future of one’s job would be expected to result in impaired performance [60]. A negative relationship between job insecurity and employee performance can also be explained by social exchange and psychological contract theories. In accordance with such theory, those employees who feel that the organization has breached the psychological contract, as a result of their job insecurity perceptions, will be more prone to suffer from emotional exhaustion and be less likely to exert themselves for the organization [44]. Additionally, social identity theory suggests a negative relationship between job insecurity and performance, as the experiencing of potential job loss erodes work-related identity [40]. However, those experiencing job insecurity may also attempt to counteract the threat to their job by working hard to show that they are worthy of keeping their employment, resulting in higher task performance and more organizational citizenship behaviors [16,17,61].

Most primary studies have found job insecurity to be negatively related to general and task performance [31,62], but some studies have found non-significant [40] or even positive associations [11,13]. The associations between job insecurity and contextual performance have also been found to be negative in most studies [63,64], although some studies have found non-significant relationships [65,66]. Job insecurity is generally found to be positively related to counterproductive work behaviors [67,68], but also negative correlations [69] and non-significant links [70] have been reported. Again, most studies have found job insecurity to be associated with poorer creativity [71,72] with some studies finding positive [73] or non-significant relationships [74]. Lastly, while most studies investigating job insecurity in relation to different indicators of safety performance have found negative associations [75,76], some have found the constructs to be unrelated [77].

In previous meta-analyses investigating the relationship between job insecurity and employee performance, the results were partly inconclusive. The first of these [19] found that job insecurity was unrelated to overall job performance, whereas a follow-up analysis six years later [9] found a moderately strong negative correlation. A meta-analysis including various work demand stressors [10] found job insecurity to be negatively related to both general performance and self-rated performance. A more recent meta-analysis on job insecurity [4], which included a range of potential consequences, found negative correlations with job performance, organizational citizenship behaviors (i.e., contextual performance), and safety behaviors, but no consistent relation with counterproductive work behavior. It should be noted that none of the previous meta-analyses included creativity as a separate outcome. Examining a greater number of studies involving job insecurity and various indicators of employee performance may provide a more robust estimate of the relationships—potentially bringing an end to the inconsistencies in previous research. Based on theoretical reasoning and the overall pattern of the findings from primary studies and meta-analyses, we made the following prediction:

**Hypothesis 1** **(H1).** Job insecurity is associated with negative performance outcomes.

### 1.4. Moderators

When the strength of associations varies between studies, the variation is usually ascribed to potential moderators [78]. This is also the case for the relationship between job insecurity and the various previously studied outcomes [79]. Previous meta-analyses on the consequences of job insecurity have tested methodological factors [4,5,9,19], demographic factors [9,19,80], and a geographical factor (region for country of data collection) [5,80] as moderators in the relationship between job insecurity and outcomes. There have also been numerous calls for the further investigation of possible moderators in the associations between job insecurity and its outcomes [6,8]. In the present study, we test two method-related factors and two macro-level indicators of social protection as potential moderators in the relationship between job insecurity and employee performance.

#### 1.4.1. Method-Related Factors

When it comes to methodological factors as moderators, one recurring topic in the job insecurity literature is whether the strength of associations varies over time [3,18,19]. It might be, for instance, that the reactions following stress appraisal [36], psychological contract breach [42], or identity threat [40] manifest at the same time as the job insecurity experience or develop over a longer period of time [19,81], thus suggesting that findings may differ between cross-sectional and longitudinal studies. The present study addresses this topic by testing possible moderator effects of cross-sectional and longitudinal associations. While a narrative review of longitudinal studies focusing on how job insecurity relates to health and well-being has found that many of the associations that have been found in cross-sectional research appear to also be valid longitudinally [3], whether this is true for the relationships between job insecurity and performance-related outcomes in particular has yet to be addressed in a meta-analytic review. Moreover, in general, it is important to explicitly compare cross-sectional and longitudinal correlations not only because of the risk for inflated effect sizes in cross-sectional designs, but also due to the fact that longitudinal evidence is more appropriate for supporting conclusions about the direction of the relationship [82]. In the few studies reporting both cross-sectional and longitudinal correlations between job insecurity and employee performance, some find stronger associations cross-sectionally than longitudinally [70,83], while others report both stronger and weaker associations between job insecurity and its outcomes longitudinally [84]. The cumulative evidence still indicates that associations between job insecurity and outcomes are stronger in cross-sectional studies. While the vast majority of studies on job insecurity have been cross-sectional, the proportion of studies with time-lagged or longitudinal designs has increased in more recent research [8]. This led us to expect the following:

**Hypothesis 2** **(H2).** The associations between job insecurity and types of employee performance are stronger in cross-sectional studies.

A second methodological issue concerns the ratings sources. The use of self-ratings has been subject to much debate [85], and many researchers have discussed the risk for mono-method bias when both the predictor and the criterion variable are based on self-ratings. This involves the risk that associations based on ratings from the same source may be inflated [21,86]. This appeared to occur in a meta-analysis investigating how various stressors relate to employee performance [10], in which role ambiguity and work–family conflict showed stronger negative correlations with self-rated performance than with performance-ratings from another source, that is, supervisors. Additionally, the study participants’ ability and/or motivation to objectively rate their own job performance were related to performance self-ratings in particular [20,87]. When one’s job is perceived to be at risk, favorably rating one’s own performance may be a strategic way to deal with the situation, for example, by trying to demonstrate one’s value to the organization [17,88]. From an analysis perspective, this might produce a weaker negative (or sometimes even a positive) relationship between job insecurity and self-rated performance, as compared to the association between job insecurity and supervisor-rated performance. However, based on research showing that ratings between constructs measured using the same source typically being more strongly correlated [21], we expect that this will be the case for job insecurity and performance ratings as well.

**Hypothesis 3** **(H3).** Job insecurity is more strongly associated with self-rated performance than with supervisor-rated performance.

#### 1.4.2. Macro-Level Indicators of Social Protection

Job insecurity has been studied across the globe, and while the consequences associated with it have generally been found to be detrimental across national contexts, the strength of associations varies. It has been argued that part of this variance may be ascribed to differences between the societal contexts wherein these reactions occur [22,24,89,90]. Such differences include, but are not limited to, characteristics of national culture, labor legislation, social security, welfare expenditure, and labor market characteristics such as unemployment rates [91,92]. These characteristics contribute to shaping a society’s level of social protection, which may influence the type and magnitude of consequences and support that employees expect in case of job loss. Previous research indicates that the generosity of welfare states may reduce levels of job insecurity [93] and has shown that employees in countries with more extensive unemployment assistance tend to perceive lower levels of job insecurity than employees in countries with less extensive unemployment assistance [94]. Moreover, more generous welfare systems, including financial support during unemployment, have been found to have positive effects on unemployment outcomes [95]. This not only indicates that strong welfare systems may reduce the levels of job insecurity, but moreover, that in welfare states with more social protection, the threat of job loss implied by job insecurity perceptions will have less severe consequences. In societies with stronger social protection, the reactions to a threat of unemployment may be less severe, because employees feel that society will help them handle the consequences. This applies to performance as well as other outcomes such as mental health, since job insecurity affects individuals by requiring resources—cognitive and emotional—to handle the threat, and fewer resources are then available to expend on job performance. This would suggest that higher levels of job insecurity are associated with lower levels of performance.

More specifically, based on theories of stress appraisal [36], psychological contract breach [42], or identity threat [40], the reactions to job insecurity can be expected to be weaker due to the feeling that, even if one risks becoming unemployed, society will help individuals to handle their unemployed circumstances [24,89,91,93]. As fewer cognitive and emotional resources are expended in dealing with threats to employment in more generous welfare states, resources can be directed towards performance-oriented behaviors, resulting in a weaker correlation between job insecurity and performance in countries characterized by stronger social protection [29,41]. Given that stronger welfare systems involve various sources of support in the event of job loss, we expect welfare regimes characterized by a high degree of social protection to buffer against negative consequences of job insecurity [89,96].

**Hypothesis 4** **(H4).** The associations between job insecurity and types of employee performance are weaker in welfare regimes characterized by a high degree of social protection compared to welfare regimes with less social protection.

Another indicator of the level of social protection in a country is union density (i.e., the proportion of union membership among a country’s employed individuals [97]). As the main goal of trade unions is to protect the interests of the employee, alleviating the risk of job insecurity has previously been upheld as an important reason for employees to join unions [25,26,98]. For instance, trade unions can negotiate better conditions—such as retraining opportunities or job search assistance—for those affected by downsizing [98,99,100]. In the event of downsizing, unions may also influence the downsizing process, by encouraging employers to work more proactively by providing key information on developments. Union membership and support from unions have been shown to have the potential to mitigate the consequences of job insecurity [26,101]. In addition, employee perceptions of procedural justice may also benefit from a strong union, as unions safeguard employee interests by ensuring that agreed upon principles and formal procedures are followed in the case of redundancies [102]. The presence of unions, as reflected in higher levels of union density, can be expected to provide employees with support if their jobs are at risk [25,26,98]. This suggests that, with a union, fewer resources need to be expended when coping with job insecurity [41], resulting in a weaker correlation between job insecurity and performance in countries characterized by higher union density [25,26,98]. More specifically, it can be anticipated that stronger social protection will make employees more able to focus on their job and less on rumination.

**Hypothesis 5** **(H5).** The associations between job insecurity and types of employee performance are weaker in countries characterized by higher union density than in countries with lower union density.

## 2. Materials and Methods 

### 2.1. Literature Search and Inclusion Criteria

We followed a three-step approach to find relevant articles for this meta-analysis on job insecurity and performance outcomes. First, text searches of several electronic databases (PsycINFO, Web of Science, and EBSCO) were conducted using the same search terms as in previous meta-analyses of job insecurity [4,9,19], comprising combinations of key terms including “job insecurity,” “job uncertainty,” “job security,” “job security satisfaction,” “satisfaction with job security,” and “employment uncertainty.” Following previous meta-analyses on job insecurity [9,19,32], the searches were restricted to peer-reviewed scientific journal articles written in English and published between 1980, and in the present case, June 2018. Second, we searched the reference lists of numerous articles, including previously published meta-analyses on the consequences of job insecurity [4,5,9,19] and literature reviews on job insecurity [3,6,24,103,104,105] to gather additional relevant articles. Third, we conducted issue-by-issue searches of several academic journals in order to find potentially relevant studies that were not identified through the electronic searches. 

The literature search yielded an initial sample of 1992 studies, of which 269 appeared to concern associations with some aspect of job performance. These studies were reviewed to assess whether they met four inclusion criteria, the same four that were used for the meta-analysis by Sverke and colleagues [19]. First, job insecurity was to be measured as a subjective perception (global, cognitive, affective, or dissatisfaction with job security). Second, the studies needed to include at least one outcome variable, in this case related to employee performance. Third, the studies should provide a correlation coefficient between job insecurity and the outcome or report other statistics that could be transformed into correlations. Fourth, the study had to be based on a working population. Studies that did not meet these inclusion criteria were excluded from the final sample. A total of 106 studies, representing 119 different samples, met the inclusion criteria and were hence included in the meta-analytic review. The studies were based on questionnaire data from a grand total of 50,928 individuals from 33 countries. A list of the included studies is presented in Supplementary Materials.

### 2.2. Coding of the Studies

Each sample was coded for sample size, type of performance-related outcome(s), correlation between job insecurity and outcome(s), reliability (Cronbach’s alpha) of job insecurity and outcome measure(s), whether the correlation was based on a cross-sectional or longitudinal sample, source of rating of performance variables (self-rated vs. supervisor-rated), and country of data collection. Only studies of quantitative job insecurity (i.e., threats to the job as such) were included. If studies reported correlations for different dimensions of such global quantitative job insecurity (such as affective and cognitive job insecurity) in relation to an outcome, we used the average correlation (and reliability estimate for job insecurity).

Regarding method-related moderators, correlations were coded as either cross-sectional or longitudinal and the outcome as self-rated or supervisor-rated. If a study reported both cross-sectional and longitudinal associations, we used the longitudinal correlation in order to include more longitudinal correlations in the analysis, thus allowing better opportunities for moderator analyses. In line with this, if a study included correlations between job insecurity and both self-rated and supervisor-rated measures of an outcome, we chose the latter. For studies reporting longitudinal correlations, we used the correlation between job insecurity measured at the first time point and the measure of the dependent variable from the second time point, regardless of the total number of data waves in the study. Studies that had a longitudinal design, but reported only correlations within time were coded as cross-sectional. 

As concerns macro-level moderators, country of data collection was, in a subsequent step, used for classifying countries based on the two macro-level indicators of social protection, namely the type of welfare regime and union density. The classification of types of welfare regimes (i.e., Scandinavian, Bismarckian, Southern European, Anglo-Saxon, Eastern European, or East Asian) was adopted from Kim et al. [24], who relied on previous research [106,107,108]. For union density, the classification was based on the average national-level unionization rate for the period 1980–2014, based on statistics reported by the Organisation for Economic Co-operation and Development, OECD [109] and the International Labour Organization, ILO [110], supplemented with information about union density in China [111] and Taiwan [112]. Countries were then categorized into low (<25%), medium (25–49%), and high (≥50%) union density for these years. Appendix A presents the welfare regime classifications of those countries which could be classified (Table A1) along with the level of union density classifications (Table A2).

The coding was conducted independently by two different pairs of coders. The average agreement between coders was 94%. The coding discrepancies were negotiated between the authors to arrive at consensus. The coding resulted in the identification of five performance outcomes (task performance, contextual performance, counterproductive work behavior, creativity, and safety performance). Table 1 provides examples of the clusters of primary study variables subsumed by each of the five overall categories and presents the average weighted reliability estimates of all variables.

### 2.3. Meta-Analytic Procedures

We used Hunter and Schmidt’s [78] meta-analysis method to calculate the meta-analytic mean correlations and the variability of associations between job insecurity and performance outcomes. Adopting a random-effect approach, we first estimated sample-size weighted average correlations ($\bar{r}$_o_) between job insecurity and each performance outcome, with a 95% confidence interval (CI) around each estimate. The CI denotes the amount of error in the estimate due to sampling error and allows for the interpretation of whether a weighted correlation differs from zero. We also estimated correlations corrected for attenuation ($\bar{r}$_c_), taking reliability in job insecurity and the performance measure into account. For those studies that did not report reliability estimates, the weighted average reliability from all studies reporting data for this variable was used. These corrected estimates were accompanied by standard deviations (*SD*) and 80% credibility intervals (CV). For each association between job insecurity and performance outcomes, we also report the number of studies (*k*) and the accumulated sample size (*N*) included in the analysis. 

In order to detect where moderators may have influenced the correlations, we relied on several sources of information. First of all, when the 80% CV, which estimates the variability in $\bar{r}$_c_, includes zero or is large, this indicates that moderators are likely present [113]. Secondly, we relied on the *Q* statistic, which is a homogeneity test indicating the variance across studies relative to sample error variance, where a significant value indicates the likely presence of moderators [114]. Finally, we tested for potential moderators using the test for the percentage of variance that can be explained by artifacts (%Var_art_). While it has been suggested that moderators are likely present when 75% or more of the variance in a weighted mean correlation is explained by artifacts, we relied on a cut-off of 60% [115], which is typically used when the correlations cannot be corrected for range restriction, as in the present meta-analysis. 

We repeated these calculations for each level of each moderator tested. Differences between moderator levels were scrutinized by examining whether the confidence intervals overlapped. In addition, we again made use of the *Q* statistic, which is similar to the analysis of variance. More specifically, differences across moderator levels were tested using the between-group homogeneity tests (*Q_B_*), where significant values indicate differences between groups. This was again supplemented by within-group homogeneity tests (*Q_W_*) and by inspecting whether the 80% CVs for the different moderator levels overlapped. The tests for moderation were performed only for those moderator variables included in a minimum of two primary studies.

## 3. Results

### 3.1. Main Effects

The first aim of the present study was to investigate how job insecurity relates to various indicators of employee performance. Table 2 presents the overall meta-analytic results for the relationships between job insecurity and each of the five identified aspects of performance. Job insecurity was negatively related to task performance ($\bar{r}$_o_ = −0.14; $\bar{r}$_c_ = −0.17) and contextual performance ($\bar{r}$_o_ = −0.14; $\bar{r}$_c_ = −0.18) and positively associated with counterproductive work behavior ($\bar{r}$_o_ = 0.11; $\bar{r}$_c_ = 0.14). In none of these cases did the CI include zero. Regarding creativity ($\bar{r}$_o_ = −0.10; $\bar{r}$_c_ = −0.10), the CI however included zero, while job insecurity evidenced a significant negative correlation with safety performance ($\bar{r}$_o_ = −0.16; $\bar{r}$_c_ = −0.18). These results provide general support for Hypothesis 1, which predicted that job insecurity is related to impaired employee performance. 

The analyses also indicated the likely presence of moderators of these overall associations. The 80% CVs were rather large and included zero. Moreover, in all cases, the *Q* statistic indicated significant heterogeneity for all relations. In addition, artifacts accounted for substantial proportions of the variance in all correlations, well beyond the cut-off of 60%. These results clearly indicate that the associations between job insecurity and employee performance are moderated by other factors.

### 3.2. The Moderating Role of Method-Related Factors

Our second aim was to investigate two method-related factors as potential moderators of the correlation between job insecurity and employee performance. Table 3 presents the results of the tests of temporal aspects (cross-sectional vs. longitudinal associations) and rating source (self-rated vs. supervisor-rated performance) for the variables where such tests were possible (i.e., when a certain moderator variable was included in at least two primary studies). 

As concerns temporal aspects, tests for differences between cross-sectional and longitudinal associations were possible for task performance, contextual performance, and counterproductive work behavior but not for creativity and safety performance, as there was not a sufficient number of longitudinal studies involving these outcomes to allow for such comparison. For task performance, there was a significant difference between these study designs regarding the magnitude of the correlations between job insecurity and the performance outcome, as indicated by the *Q_B_* statistic. The association ($\bar{r}$_o_ = −0.16; $\bar{r}$_c_ = −0.19) was only significant for the cross-sectional correlations, whereas the CI for the longitudinal correlation included zero. In terms of contextual performance, neither the correlation for cross-sectional designs ($\bar{r}$_o_ = −0.15; $\bar{r}$_c_ = −0.18) nor that for longitudinal designs ($\bar{r}$_o_ = −0.08; $\bar{r}$_c_ = −0.09) had CIs including zero, and there was no significant difference found between study designs. The same was true for counterproductive work behavior, where both the cross-sectional ($\bar{r}$_o_ = 0.10; $\bar{r}$_c_ = 0.13) and the longitudinal ($\bar{r}$_o_ = 0.16; $\bar{r}$_c_ = 0.19) correlations had CIs that did not include zero. While there was little variation in the longitudinal associations for contextual performance and counterproductive work behavior, the other associations indicated additional heterogeneity. Taken together, these results provide only partial support for Hypothesis 2, which proposed that the magnitude of associations is stronger in cross-sectional studies. Specifically, the hypothesis was only supported in regard to task performance, whereas the results concerning contextual performance and counterproductive work behavior indicated no differences between cross-sectional and longitudinal studies in the associations between job insecurity and these performance outcomes. 

In regards to rating source, moderator analyses were possible only for task performance and contextual performance (Table 3). There was a significant difference between self- and supervisor-ratings for task performance, as indicated by the *Q_B_* test, where the relation was somewhat stronger for self-ratings ($\bar{r}$_o_ = −0.15; $\bar{r}$_c_ = −0.18) than for supervisor-ratings ($\bar{r}$_o_ = −0.09; $\bar{r}$_c_ = −0.11). There was no significant difference between rating sources concerning contextual performance, where the association with job insecurity was negative for both self-ratings ($\bar{r}$_o_ = −0.14; $\bar{r}$_c_ = −0.18) and supervisor-ratings ($\bar{r}$_o_ = −0.15; $\bar{r}$_c_ = −0.19). In both cases, however, the analyses still indicated heterogeneity according to the *Q_W_* tests. While the results regarding task performance provide partial support for Hypothesis 3—that job insecurity evidences stronger associations with self-rated performance—the finding that the association with contextual performance did not differ between self- and supervisor-ratings partially contradicts this postulation.

### 3.3. The Moderating Role of Macro-Level Indicators of Social Protection

The third aim of the present study was to investigate whether two macro-level indicators of social protection moderate the association between job insecurity and employee performance. The results of these analyses are presented in Table 4, which presents the determined types of welfare regimes (Scandinavian, Bismarckian, Southern European, Anglo-Saxon, Eastern European, and East Asian) as well as the union density levels of high (≥50%), medium (25–49%), or low (<25%). Again, the results are only presented for those moderator variable levels included in at least two primary studies. 

There were significant differences between the types of welfare regimes as concerns the associations of job insecurity with all of the employee performance outcomes (excluding creativity, where no such comparisons were possible), as indicated by the *Q_B_* tests (see Table 4). When it comes to task performance, there were negative correlations for all of the types of welfare regimes that could be included in the analysis (there were not enough studies from Eastern European or East Asian countries to include these areas in the analysis). The correlation was weakest in Scandinavian countries ($\bar{r}$_o_ = −0.04; $\bar{r}$_c_ = −0.04) and strongest in Southern European countries ($\bar{r}$_o_ = −0.20; $\bar{r}$_c_ = −0.25), with Anglo-Saxon ($\bar{r}$_o_ = −0.12; $\bar{r}$_c_ = −0.14) and Bismarckian countries ($\bar{r}$_o_ = −0.18; $\bar{r}$_c_ = −0.22) in-between. The correlation between job insecurity and contextual performance was not significant in Bismarckian countries; the negative association was strongest in East Asian countries ($\bar{r}$_o_ = −0.25; $\bar{r}$_c_ = −0.31), followed by Southern European ($\bar{r}$_o_ = −0.15; $\bar{r}$_c_ = −0.19) and Anglo-Saxon countries ($\bar{r}$_o_ = −0.14; $\bar{r}$_c_ = −0.17) (Scandinavian and Eastern European regimes could not be included in this analysis). For counterproductive work behavior, comparisons were only possible between Southern European countries, where there was a positive correlation ($\bar{r}$_o_ = 0.24; $\bar{r}$_c_ = 0.30), and Anglo-Saxon countries for which the association was non-significant. The results for safety performance followed a slightly different pattern: The negative correlation was strongest in Scandinavian ($\bar{r}$_o_ = −0.20; $\bar{r}$_c_ = −0.23) and Anglo-Saxon countries ($\bar{r}$_o_ = −0.17; $\bar{r}$_c_ = −0.21) and weakest in Southern European countries ($\bar{r}$_o_ = −0.05; $\bar{r}$_c_ = −0.06). These results provide partial support for Hypothesis 4, which predicted that the associations between job insecurity and employee performance would be weaker in welfare regimes characterized by high social protection (e.g., Scandinavian and Bismarckian) and stronger in regimes where social protection is lower (e.g., Southern European and East Asian).

In terms of our second indicator of social protection, there was a significant difference for task performance, where the correlation was weaker in countries with medium union density ($\bar{r}$_o_ = −0.10; $\bar{r}$_c_ = −0.12) and stronger in countries with high ($\bar{r}$_o_ = −0.15; $\bar{r}$_c_ = −0.17) and low ($\bar{r}$_o_ = −0.15; $\bar{r}$_c_ = −0.18) union density. There were no significant differences between countries with different levels of union density as concerns contextual performance ($\bar{r}$_o_ ranging from −0.11 to −0.14; $\bar{r}$_c_ ranging from = −0.15 to −0.17) or counterproductive work behavior ($\bar{r}$_o_ = 0.15; $\bar{r}$_c_ ranging from 0.17 to 0.18). However, there was a significant difference in creativity, as indicated by the *Q_B_* test. The correlation was negative in countries with high union density ($\bar{r}$_o_ = −0.09; $\bar{r}$_c_ = −0.10), whereas the seemingly positive association in countries with medium union density was non-significant (and there were not enough studies for the low union density category). There was a significant difference also with respect to safety performance. As indicated by the CIs, job insecurity evidenced a significantly stronger negative correlation with safety performance in countries with high union density ($\bar{r}$_o_ = −0.20; $\bar{r}$_c_ = −0.23) than in countries with low union density ($\bar{r}$_o_ = −0.10; $\bar{r}$_c_ = −0.12), with effects sizes for medium union density ($\bar{r}$_o_ = −0.19; $\bar{r}$_c_ = −0.23) being comparable to those for high union density. These results are not in line with Hypothesis 5, which predicted that the associations between job insecurity and employee performance would be weaker in countries characterized by higher union density.

## 4. Discussion

The present meta-analysis combined research from 119 samples from 106 studies on the relationships between job insecurity and five types of employee performance—task performance, contextual performance, counterproductive work behavior, creativity, and safety performance. In addition, we conducted moderator analyses to better understand variations in the strength of the associations between job insecurity and these types of employee performance. The moderator variables were chosen to enable the investigation of factors which in previous research have been assumed to affect this type of relationship, but which have not been extensively tested in a meta-analytic context. Two of the moderators are related to methodological issues: Study design (cross-sectional compared to longitudinal) and source of employee performance ratings (self-rated compared to supervisor-rated). The other two moderators concerned aspects of the national context of the research populations, focusing on indicators of social protection, namely, type of social welfare regime (comparing six different categorizations) and union density (low, medium, and high). 

### 4.1. Job Insecurity and Employee Performance

The first aim of the present study was to meta-analytically investigate how job insecurity relates to different types of employee performance. We hypothesized that job insecurity would be associated with negative performance outcomes (i.e., lower task and contextual performance, higher counterproductive work behavior, and lower creativity and safety performance). 

A pattern emerged in the aggregate correlations between job insecurity and types of employee performance, as the associations consistently indicated that job insecurity is related to impaired performance—the only exception being creativity. The relationships found between job insecurity and the other four types of employee performance confirm the substance of what could be expected according to arguments based on stress theories [36,41], social exchange theories [42], and identity theories [40]: Employees who experience job insecurity are less likely to make an effort to support organizational goals. The results are consistent with much of the research on job insecurity and how it relates to various performance outcomes, including two meta-analyses on job insecurity that included job performance [4,9]. The finding that job insecurity appears to be detrimental for employee performance is also contrary to those studies suggesting that experiences of job insecurity will make employees work harder [16,17]. The fact that we did not find any relationship between job insecurity and creativity could be attributed to several different factors, for example, that while job insecurity is associated with other types of performance [69,74], it is not related to creative and innovative performance. Another factor may be that other mechanisms are linking job insecurity to creativity, such as intrinsic motivation [116] and threat rigidity [117,118]. However, most primary studies investigating this relationship report a negative association between job insecurity and creativity and innovative behaviors [66,119]. Hence, the non-significant meta-correlation between job insecurity and creativity found in the present study may also be attributable to a lack of power due to the small number of studies (*k* = 10) examining this relationship.

The present meta-analysis only examined linear relationships, and did not explore the possibility of these relationships being non-linear. Studies that present curvilinear associations have indicated that job insecurity is negatively related to performance, but that this relationship is weaker when job insecurity is high [120,121]. In cases where a high level of job insecurity is found to be more weakly associated with performance, it has been attributed to these individuals being better able to handle the pressure that accompanies high job insecurity [122]. As only a limited number of studies have explored the possibility of there being non-linear relationships between job insecurity and outcomes such as employee performance, it was not considered worthwhile to aggregate the results of these studies in the present meta-analysis, but this is an avenue that should be explored in future research when appropriate. 

Since job insecurity in the present meta-analysis was treated as a global construct reflecting threats to the job itself (not including valued job features, i.e., qualitative job insecurity), no differentiation occurred among the different operationalizations of job insecurity from the primary studies included. To be sure, previous research, including a recent meta-analysis [4], has indicated that there may be differences between cognitive and affective job insecurity in regard to how they relate to various outcomes, and that qualitative and quantitative job insecurity may relate differently to outcomes [122], but these differences have not have been tested extensively in relation to performance yet. Our meta-analysis provides a first step in confirming the overall negative impact on performance that job insecurity appears to have. Comparing different dimensions of job insecurity to global measures of job insecurity in future research will elucidate which operationalizations explain the most variance in employee performance. 

The present meta-analytic findings indicate that job insecurity is associated with lower task performance, lower contextual performance, higher counterproductive behavior, and lower safety performance (with the association with creativity not being significant), thus possibly contributing to bringing an end to inconsistencies among previous studies regarding such associations. However, for all main effects there were indications that they were affected by potential moderators. The recent job insecurity literature has increasingly called for additional research on mechanisms in the relation between job insecurity and its outcomes [3,123]—in terms of both mediators [124] and moderators [6,104]. In an attempt to add to this literature, we also tested several moderators of the associations between job insecurity and the types of employee performance. 

### 4.2. The Role of Temporal Aspects and Source of Ratings

The second aim of the present meta-analysis was to test two method-related factors as potential moderators in the associations between job insecurity and types of employee performance, namely, temporal aspects and source of performance ratings. We hypothesized that the associations with performance would be stronger in cross-sectional, as compared to longitudinal designs (Hypothesis 2) and that job insecurity would be more strongly associated with self-rated performance than with supervisor-rated performance (Hypothesis 3).

The results of the moderator analyses focusing on temporal aspects of study design indicated that only the relationship between job insecurity and task performance differed between types of study design: the association was stronger in cross-sectional studies and non-significant in longitudinal studies. Hypothesis 2 thus only received partial support. These results may indicate that task performance is affected by job insecurity in the short term, but not in the long term, and that the effects of job insecurity on performance are synchronous and may not be cumulative. Lack of cumulative effects of job insecurity on task performance could be attributed to the fact that task performance is necessary for employees to keep their job in the long term, but that it is affected by the strain of job insecurity in the short term. 

However, for the other two outcomes where such moderator tests were possible (i.e., contextual performance and counterproductive work behaviors), the association with job insecurity was significant both cross-sectionally and longitudinally, suggesting that the experiencing of job insecurity can also have detrimental effects over time. Such delayed detrimental effects may be due to a sense that the organization has breached the psychological contract, resulting in a lack of trust in the organization [46] that may manifest itself as a decrease in pro-organizational discretional behaviors or as an increase in negative behaviors directed at the source of the breach. In addition, the finding that there was no difference in magnitude between cross-sectional and longitudinal correlations for contextual performance and counterproductive work behaviors also suggests that cross-sectional associations may be a fairly accurate representation of how job insecurity relates to these types of performance. It should be noted, however, that the number of studies reporting longitudinal correlations between job insecurity and both contextual performance (*k* = 2) and counterproductive work behaviors (*k* = 3) was quite small, and that the effect sizes varied considerably among the included primary studies. More longitudinal research on these relationships would enable more robust meta-analytical tests of the associations between job insecurity and performance over time. 

The moderator analysis focusing on source of performance ratings partially supported Hypothesis 3, as job insecurity was found to relate more strongly to self-ratings of task performance than to supervisor ratings. This is partially in line with previous meta-analytic findings where work stressors evidenced stronger negative relationships with self-rated as compared to supervisor-rated performance [10]. However, there was no difference between rating sources for the relationship between job insecurity and contextual performance. This finding contributes to the job insecurity literature by suggesting that job insecurity may be detrimental to this type of performance, and that the relationship is evident regardless of whether performance is rated by employees or by supervisors. Previous research on the effect of rating source on the correlation between overall performance and contextual performance has indicated that self-ratings are more strongly correlated than constructs rated by other sources [125], but the results pertaining to contextual performance in the present study did not follow this pattern. Considering that the correlation between self- and supervisor-ratings of performance in general tends to be quite weak, with a corrected meta-analytic correlation of 0.22 [20], and that little research exists on whether the relationships between job insecurity and various types of performance differ depending on the source of performance ratings, future research investigating how job insecurity relates to both self-rated and supervisor-rated performance appears highly warranted. Future research would also benefit from using supervisor-ratings of types of performance other than task performance and contextual performance, which were the only dimensions for which this moderator analysis could be performed.

### 4.3. The Role of Welfare Regimes and Union Density

The third aim of this meta-analysis was to test whether two macro-level indicators of social protection (type of welfare regime and union density) moderate the associations between job insecurity and types of employee performance. 

The results of the moderator analyses focusing on the type of welfare regime provided partial support for Hypothesis 4, which predicted that the associations between job insecurity and employee performance would be weaker in welfare regimes characterized by high social protection (e.g., Scandinavian and Bismarckian) and stronger in regimes where social protection is lower (e.g., Southern European and East Asian). While it was not possible to include all types of welfare regimes as moderators in all insecurity–performance associations, the comparisons still indicated general tendencies in so far as the associations with task performance and counterproductive work behaviors were weaker in welfare regimes characterized by higher social protection, and the negative association with contextual performance was stronger in regimes considered to provide lower social protection. These findings are in line with the notion that the generosity of welfare states may reduce levels of job insecurity [92] and protect against negative consequences of unemployment [94], and thus may also buffer against a negative impact of job insecurity. However, the finding that job insecurity evidenced stronger negative associations with safety performance in regimes with higher social protection (Scandinavian and Anglo-Saxon) than in those characterized by lower social protection (Southern European countries) was unexpected. Employees in regimes with higher social protection may have come to expect a high degree of employment certainty and may thus react more negatively when experiencing job insecurity, as exhibited in the stronger negative associations with safety performance. This explanation is tentative and the results call for additional research regarding the role of welfare regimes. 

The results of the moderator analyses focusing on union density were not in line with Hypothesis 5, which predicted that the associations between job insecurity and employee performance would be weaker in countries characterized by higher union density. More specifically, the finding that there were no differences between countries with high, medium, and low union densities as concerns the associations of job insecurity with contextual performance and counterproductive work behavior is contrary to the assumption that union membership may buffer against negative consequences of job insecurity [26,99]. Most surprising was the finding that the negative association with safety performance was stronger in countries with high and medium union density as compared to countries with low union density. It was also unexpected that the association with task performance was higher in countries with both high and low union density, as compared to those with medium union density. One possible explanation for the finding that reactions were not weaker in countries characterized by high union density may be that higher union density can lead to higher expectations from, and attentiveness to, the psychosocial work environment. Hence, union density as such might not constitute social protection for employees. Rather, awareness and employee expectations of employment security and safety are heightened when trade union membership is the ‘norm’ in society. 

Taken together, the results concerning indicators of social protection lend some support to the argument that characteristics of the welfare regime and of unionization are of relevance for the performance-related consequences of job insecurity. One point of discussion that is often raised in connection with country comparisons is that any typology of them will utilize fairly broad clusters of countries with substantial variance within each cluster of countries on a number of parameters [126]. It could be that other ways of classifying countries may tell a different story. Some studies have, for instance, focused on different indicators of national culture, such as uncertainty avoidance, or on differences between individualist and collectivist cultures [92,127,128]. For instance, it is plausible that countries characterized by high uncertainty avoidance built their social safety nets in order to avoid employment uncertainty or to reduce the negative consequences of job insecurity [128,129]. Future research may benefit from exploring other types of macro-level indicators of social protection, such as cultural indicators (e.g., uncertainty avoidance) or indicators of labor market policies (e.g., public expenditure in active labor market policies). It is also possible that the level of social protection in a society may serve to reduce the levels of job insecurity perceived by employees, rather than acting as a buffer in the association between job insecurity and employee performance outcomes [93]. 

### 4.4. Methodological Considerations

Meta-analysis is a powerful tool for integrating empirical studies relating job insecurity to employee performance, but similar to other meta-analyses, our study has some limitations, which need to be acknowledged, and if possible, addressed in future research. These arise from the challenging choices involved in study inclusion, the application of concepts, and the integration of findings from different types of studies. One obvious limitation is that it is possible that the literature search missed out on some relevant studies despite our use of several search approaches to identify all studies that belong in the review. 

Another limitation concerns the definitions and operationalizations of the job insecurity construct, which varied between the studies included in the meta-analysis, thus resulting in studies using different measures to capture job insecurity being combined in the analyses. The primary studies included both single-item measures and multi-item scales, with some focusing on the affective experience of job insecurity (i.e., worry about job loss), others on the cognitive dimension (i.e., the likelihood of job loss) or overall job insecurity, and yet others on employees’ satisfaction with job security. A previous meta-analysis on job insecurity [19] found that the associations between job insecurity and its outcomes are stronger in studies utilizing scales containing multiple items, as compared to those relying on single items. It has also been demonstrated that measures of a global experience of job insecurity are more stable over time as compared to measures focusing on a specific dimension of the construct [130]. A recent meta-analysis, which distinguished between cognitive and affective job insecurity [4], found affective job insecurity to have stronger relations to outcomes as compared to cognitive insecurity, and also that affective job insecurity mediates the associations between cognitive job insecurity and outcomes. While the present meta-analysis focused on threats to the job as such (i.e., quantitative job insecurity), it has been found that the relationships with outcome variables differ between quantitative (threats to the job as such) and qualitative (threats to valued job features) job insecurity [33,122,131], and once there are enough studies on the qualitative dimension, future meta-analyses could also include this dimension and explore if consequences differ between quantitative and qualitative job insecurity. We elected to include studies with different operationalizations in order to meta-analyze as extensive a pool of studies on our topic as possible. How to better handle the integration of different operationalizations should be one of the objectives of future meta-analyses in order to fine tune the combined use of an array of studies with varying approaches on the same question. 

Similarly, different types of employee performance were categorized into broader constructs in the present study. To our knowledge, this is the first meta-analysis focusing solely on the job insecurity–employee performance association, synthesizing this area of research. However, this method of categorizing performance means that there is some variation in the constructs included in the broader categories. This may present a challenge to the interpretation of the results, and to the drawing of conclusions on practical implications. Therefore, it would be useful for future research to focus on investigating the relationships between job insecurity and more fine-grained sub-dimensions of job performance, such as organizational citizenship behaviors directed towards individuals or the organization [53] or employee voice [99,132]. This may provide valuable insights for the practitioner community as well as contribute to theory development in job insecurity research.

A strength of the present meta-analysis concerns the comparison between cross-sectional and longitudinal associations of job insecurity with the performance outcomes. While the correlation between job insecurity and task performance was stronger in studies with a cross-sectional design, similar magnitudes of association emerged for two of the three outcomes among cross-sectional as well as longitudinal designs, where such moderator analyses were possible (i.e., contextual performance and counterproductive work behavior). While this could be taken to suggest that these associations are also valid over time, it does not indicate the direction of the relationships, nor allow for conclusions about causality. However, there is theoretical and empirical support for the assumed direction of the relationship, such that job insecurity predicts its postulated outcomes rather than the other way around, which has been supported in several studies [38,133]. There has been less research exploring the relationships between job insecurity and types of employee performance longitudinally, especially testing for reversed causality, and future research may consider exploring these associations using cross-lagged study designs. 

In addition, the longitudinal studies incorporated in the present meta-analysis investigated the relationships between job insecurity and types of employee performance over different time lags. Little is known about whether the associations differ between job insecurity and types of employee performance depending on the time lag [81,134]. In order to support theory development regarding how long it may take before consequences of job insecurity emerge, future studies should systematically test the associations over different time lags. The length of the time lag could then be entered as a moderator in future meta-analyses, in order to allow for the testing of how the impact of job insecurity varies depending on the time between the measurements of job insecurity and of its outcomes [135]. It is also plausible that individuals who experience job insecurity to varying degrees over time react differently to such experiences [136,137], thus calling for a more person-oriented approach that allows for studying how different patterns of accumulated experiences of job insecurity over time relate to different types of consequences [138].

A potential study design limitation concerns our use of cross-sectional studies, as it has been pointed out that such studies may overestimate the associations between job insecurity and its outcomes due to common method variance [21,86]. To address this, we explored the difference in associations between studies using self-reported ratings of performance and supervisor-ratings of performance. The results indicated that the association was slightly stronger between job insecurity and self-rated task performance than between job insecurity and supervisor-rated task performance. However, there was no significant difference regarding contextual performance, suggesting that common method variance may not have inflated the associations in the present study, which is in line with findings showing that common method variance does not distort associations between constructs sharing the same measure [139]. 

Finally, previous meta-analyses indicate that culture may be an important factor in how job insecurity relates to potential antecedents [32] as well as outcomes [80]. A key aspect of the countries encompassed by the meta-analysis in terms of our investigation is their degree of social protection, which we based on previous research [24] and categorized into types of welfare regimes according to regional and cultural zone trends. While we mainly found support for the notion that welfare regimes characterized by stronger social protection acted as a buffer in the associations between job insecurity and employee performance, union density, the other macro-level indicator of social protection included generated mixed results. It is possible that the coding of union density in the present meta-analysis may have been too blunt of a measure of union protection, thus contributing to the mixed findings. For instance, while union density was coded based on the average unionization rate during a long period of time (1980−2014), it may be more appropriate to instead rely on the unionization rate at the time of data collection for each included study—although this presents difficulties as the year of data collection is not always reported in primary studies. In addition, not all countries are covered by the classification of welfare regimes used [24]. Future meta-analyses may therefore wish to utilize other indicators of social protection which have been investigated in previous job insecurity research, such as uncertainty avoidance [62,129], unemployment benefits [94], employment protection legislation, and public expenditure on active labor market activities [93]. In addition, while the fact that we did not include unpublished studies may potentially have affected our findings, previous research suggests that there are no substantial differences in the magnitude of correlations in published, as compared to non-published studies [140], and has shown that failing to include non-published studies is not a serious threat to the validity of the conclusions drawn based on meta-analytical relationships.

### 4.5. Theoretical and Practical Implications

The results of the present meta-analysis appear to confirm the expected associations between job insecurity and negative performance outcomes, consistent with much of previous research. More specifically, our results replicate previous meta-analyses that have found that job insecurity is detrimental for employee performance [4,9], and also extend existing research by investigating a wider range of performance outcomes (task performance, contextual performance, counterproductive work behavior, creativity, and safety performance). The findings that job insecurity was associated with lower levels of task, contextual, and safety performance and higher levels of counterproductive behavior (but not with creativity on a general level) diverge from those of certain other studies which suggest that job insecurity will lead to employees working harder [16,17]. Rather, our results are in line with theories suggesting that job insecurity is associated with impaired performance due to it being a stressor [15] and that it constitutes a breach of the psychological contract [42] or a threat to one’s identity [40]. 

For future research, we would recommend that longitudinal research is prioritized. Studies conducted using a within-person design, allowing for the study of the process of how job insecurity relates to types of employee performance, will further the understanding of job insecurity and its outcomes. There are currently very few studies which differentiate between group-level and individual-level differences [38], and more research is needed to better understand within-person processes in this context in particular. There is also a need for additional research using a person-oriented approach, thus allowing for the investigation of how varying experiences of job insecurity over time relate to potential consequences [137,138]. Research using a person-oriented approach, where groups of individuals with different experiences of job insecurity are investigated—particularly with regard to various types of job insecurity and job insecurity experiences over time—may be especially relevant for identifying those groups that are at most risk from the harmful consequences of job insecurity [138]. 

Research in this area could also benefit from an exploration of other types of rating sources of job insecurity outcomes. In the present meta-analysis, only two types of employee performance could be included in the moderator analysis testing for differences between self-ratings and supervisor-ratings (i.e., task performance and contextual performance), due to the lack of studies. Performance is a complex construct to measure, and it is often recommended that a multi-rater approach be used in order to capture different aspects of performance [141].

For organizations, the results have implications concerning the impact of an insecure environment on performance: Employees cannot be expected to perform at the same level when experiencing job insecurity. We would suggest that it is in the interest of organizations to prevent the experiencing of job insecurity among employees. Not only will this prevent employees experiencing a significant stressor, it will also ensure that employees are better able to work towards organizational goals. 

The present findings also have potential implications for national-level policies. Most importantly, job insecurity, in relation to most but not all performance variables, was associated with less detrimental performance outcomes in welfare regimes characterized by high rather than low social protection. This indicates that national policies and legislation that allow stronger protection in cases of potential job loss may protect not only employees from more negative consequences, but may also end up benefitting organizations in terms of less poor employee performance outcomes. However, the findings regarding union density as an indicator of social protection produced results that are more ambiguous, and less consistent with the expectation that countries with higher unionization would have a weaker negative associations between job insecurity and employee performance. It may be that having a high degree of unionization in a country may deter negative outcomes in terms of employee performance from emerging [26,98,102,142]. However, the results may also be explained by employees in countries characterized by a high unionization rate potentially being more vigilant toward detrimental working conditions, including the risk of job loss [99,143], which may result in stronger negative reactions to job insecurity in these countries [99]. Overall, the mechanism by which social protection in a country relates to job insecurity and its impact on outcomes deserves further research. For example, exploring how individuals perceive the welfare system in their country would be efficient in protecting them should they lose their job, and relating these perceptions to their reactions to job insecurity may provide more specific information about whether and how welfare systems buffer against the negative effects of job insecurity.

## 5. Conclusions

The present study aimed at bringing some clarity among research on the relationship between job insecurity and employee performance by meta-analytically investigating how job insecurity relates to five different types of employee performance (i.e., task performance, contextual performance, counterproductive work behavior, creativity, and safety performance). The results are consistent with previous meta-analyses [4,9,10], but extend these studies by including a greater number of performance outcomes, as well as a greater number of studies. The results provide evidence that job insecurity is associated with impaired task and contextual performance, higher levels of counterproductive work behaviors, and poorer safety performance. Thus, job insecurity may jeopardize organizational goal attainment in that employees who are worried about the future of their job are less able to perform tasks. Additionally, job insecurity may lead to a less positive work environment as contextual performance may be negatively affected, and counterproductive work behaviors may increase. Job insecurity may even contribute to a less safe work environment as employees experiencing job insecurity appear to engage less in safety-related behaviors. The evidence provided should encourage organizations to aim to prevent job insecurity among their employees. 

Our moderator analyses of method-related factors showed that the associations between job insecurity and employee performance vary depending on the type of study design used (cross-sectional compared to longitudinal), with cross-sectional associations being somewhat stronger. However, the differences between study designs were not as consistent across types of performance, or as large as expected; for two of the types of employee performance, there were very few longitudinal studies available, suggesting that there is a need for more longitudinal research to allow for more robust meta-analytic estimates. Overall, the variation in the results point to the need for a more detailed investigation of the temporal nature of the associations between job insecurity and performance outcomes. Such research would benefit from focusing on within-person processes to explore how job insecurity experiences evolve over time and how outcomes are affected as a result of these changes. 

For our other method-related factor—source of performance ratings—the results were mixed; a stronger association emerged between job insecurity and self-rated task performance compared to supervisor-rated task performance, while no moderating effect of source of performance ratings was found for the association between job insecurity and contextual performance. The results suggest that self-ratings and supervisor-ratings of performance may be similarly related to job insecurity, but more studies that incorporate various methods of measuring outcomes of job insecurity are needed to enable further analysis of how job insecurity relates to outcomes that are not self-rated. 

The moderator analyses focusing on whether the effect of job insecurity might be buffered by social protection factors also provided mixed results. In terms of social welfare regimes, there was some support for our expectation that employees residing and working in a country with more protective welfare systems react less negatively to job insecurity in terms of performance, as this was the case for task performance and counterproductive work behaviors. However, the finding that the association between job insecurity and safety performance was stronger in welfare regimes with high social protection was unexpected and highlights an important avenue for future research. 

Union density did not seem to function as a buffer in the studies included in the moderator analyses investigating contextual performance and counterproductive work behaviors. The results regarding task performance, creativity, and safety performance, however, were contrary to expectations, in that stronger negative reactions to job insecurity were found among countries with higher union density. Further investigation is needed: Measuring unionization rates at the organizational level rather than the country level, for example, may yield more specific information about the role of unionization for individuals, and more locally valid results. 

The present meta-analysis contributes to the understanding of job insecurity by summarizing results from a wide variety of studies. The meta-analysis also provides an important stepping stone for future research exploring job insecurity and its outcomes. 

## Figures and Tables

**Table 1:** Variables Included in the Meta-Analysis, Examples of Aspects Covered by the Primary Studies, and Average Weighted Reliability (Cronbach’s alpha) across Samples.

Variable	Examples of Included Aspects	Average α
Job insecurity	(Overall) job (in)security, fear/worry of job loss (affective), probability of job loss (cognitive), satisfaction with job security (r)	0.84
Task performance	Performance, job/task performance, in-role behavior/performance, productivity	0.83
Contextual performance	Organizational citizenship behavior, helping behavior, knowledge-sharing behavior, information sharing, extra-role behavior, sportsmanship	0.81
Counterproductive work behavior	Counterproductive work behavior, counterproductivity, work withdrawal, deviant behavior, non-compliant job behaviors	0.78
Creativity	Creativity, creative performance, innovative work behavior, organizational innovation, idea implementation	0.90
Safety performance	Risk taking behavior (r), (behavioral) safety compliance, safe working, physical/psychosocial safety behavior	0.84

(r) = reverse coded.

**Table 2:** Main effects.

Outcome	*k*	*N*	$\bar{r}$_o_	95% CI	$\bar{r}$_c_	*SD*	80% CV	*Q*	%Var_art_
Task performance	53	21,461	−0.14	[−0.17, −0.11]	−0.17	0.14	[−0.33, −0.00]	310.17 ***	83.24
Contextual performance	37	11,031	−0.14	[−0.19, −0.10]	−0.18	0.16	[−0.37, −0.00]	201.22 ***	82.11
Counterproductive work behavior	19	7219	0.11	[0.03, 0.19]	0.14	0.22	[−0.14, 0.41]	240.51 ***	92.52
Creativity	10	5964	−0.10	[−0.21, 0.01]	−0.10	0.19	[−0.34, 0.13]	191.33 ***	95.30
Safety performance	31	18,680	−0.16	[−0.21, −0.10]	−0.18	0.19	[−0.43, 0.06]	522.36 ***	94.26

*Note*. *k* = number of samples; *N* = accumulated sample size; $\bar{r}$_o_ = sample-size weighted mean correlation; CI = confidence interval; *SD* = standard deviation; $\bar{r}$_c_ = correlation corrected for measurement error; CV = credibility interval; *Q* = homogeneity statistic Q (chi-square test of heterogeneity); %Var_art_ = proportion of variance explained by artifacts. *** *p* < 0.001.

**Table 3:** Meta-analytic results by method-related moderators.

Outcome	*k*	*N*	$\bar{r}$_o_	95% CI	$\bar{r}$_c_	*SD*	80% CV	*Q_B_* *Q_W_*
*Cross-Sectional and Longitudinal Associations*								
**Task performance**								37.03 ***
Cross-sectional	43	18,287	−0.16	[−0.19, −0.12]	−0.19	0.13	[−0.34, −0.04]	240.70 ***
Longitudinal	10	3174	−0.04	[−0.10, 0.02]	−0.04	0.12	[−0.17, 0.08]	32.36 ***
**Contextual performance**								2.15
Cross-sectional	35	10,517	−0.15	[−0.19, −0.10]	−0.18	0.16	[−0.38, −0.00]	199.25 ***
Longitudinal	2	514	−0.08	[−0.10, −0.06]	−0.09	0.02	[−0.09, −0.09]	0.10
**Counterproductive work behavior**								3.38
Cross-sectional	16	6197	0.10	[0.01, 0.20]	0.13	0.24	[−0.17, 0.43]	234.70 ***
Longitudinal	3	1022	0.16	[0.12, 0.21]	0.19	0.04	[0.19, 0.19]	1.54
*Self-Rated and Supervisor-Rated Performance Outcomes*								
**Task performance**								9.97 **
Self-Rated	38	17,642	−0.15	[−0.19, −0.11]	−0.18	0.14	[−0.35, −0.01]	267.06 ***
Supervisor-Rated	15	3819	−0.09	[−0.14, −0.05]	−0.11	0.11	[−0.22, −0.01]	33.75 **
**Contextual performance**								0.80
Self-Rated	21	6836	−0.14	[−0.20, −0.08]	−0.18	0.17	[−0.38, −0.02]	136.48 ***
Supervisor-Rated	16	4195	−0.15	[−0.21, −0.09]	−0.19	0.14	[−0.35, 0.03]	63.82 ***

*Note*. *k* = number of samples; *N* = accumulated sample size; CI = confidence interval; $\bar{r}$_o_ = sample-size weighted mean correlation; $\bar{r}$_c_ = correlation corrected for measurement error; *SD* = standard deviation; CV = credibility interval; *Q_B_* = homogeneity statistic *Q* between groups; *Q_W_* = homogeneity statistic *Q* within groups. ** *p* < 0.01, *** *p* < 0.001.

**Table 4:** Meta-analytic results by macro-level moderators.

Outcome	*k*	*N*	$\bar{r}$_o_	95% CI	$\bar{r}$_c_	*SD*	80% CV	*Q_B_* *Q_W_*
*Type of Welfare Regime*								
**Task performance**								37.18 ***
Scandinavian	3	1725	−0.04	[−0.17, −0.10]	−0.04	0.14	[−0.20, 0.13]	25.21 ***
Bismarckian	10	5790	−0.18	[−0.27, −0.10]	−0.22	0.15	[−0.40, −0.03]	105.36 ***
Southern European	3	1124	−0.20	[−0.24, −0.17]	−0.25	0.04	[−0.25, −0.25]	1.10
Anglo-Saxon	22	6878	−0.12	[−0.16, −0.08]	−0.14	0.12	[−0.26, −0.02]	67.40 ***
Eastern European	−	−	−	−	−	−	−	−
East Asian	−	−	−	−	−	−	−	−
**Contextual performance**								4.91 ***
Scandinavian	−	−	−	−	−	−	−	−
Bismarckian	4	602	−0.18	[−0.53, 0.18]	−0.23	0.43	[−0.78, 0.32]	83.52 ***
Southern European	2	785	−0.15	[−0.23, −0.06]	−0.19	0.07	[−0.24, −0.15]	3.19
Anglo-Saxon	9	2213	−0.14	[−0.22, −0.05]	−0.17	0.17	[−0.36, 0.01]	38.58 ***
Eastern European	−	−	−	−	−	−	−	−
East Asian	2	477	−0.25	[−0.30, −0.19]	−0.31	−	[−0.31, −0.31]	.85
**Counterproductive work behavior**								14.69 ***
Scandinavian	−	−	−	−	−	−	−	−
Bismarckian	−	−	−	−	−	−	−	−
Southern European	3	988	0.24	[0.20, 0.28]	0.30	0.03	[0.30, 0.30]	1.14
Anglo-Saxon	5	1287	0.08	[−0.04, 0.20]	0.10	0.16	[−0.08, 0.28]	22.75 ***
Eastern European	−	−	−	−	−	−	−	−
East Asian	−	−	−	−	−	−	−	−
**Safety performance**								26.38 ***
Scandinavian	3	2431	−0.20	[−0.22, −0.17]	−0.23	0.01	[−0.23, −0.23]	0.35
Bismarckian	−	−	−	−	−	−	−	−
Southern European	3	2034	−0.05	[−0.07, −0.04]	−0.06	0.02	[−0.06, −0.06]	0.44
Anglo-Saxon	8	2928	−0.17	[−0.24, −0.09]	−0.21	0.12	[−0.34, −0.08]	31.93 ***
Eastern European	−	−	−	−	−	−	−	−
East Asian	−	−	−	−	−	−	−	−
*Union Density*								
**Task performance**								7.27 *
High (≥50%)	10	6691	−0.15	[−0.23, −0.07]	−0.17	0.15	[−0.36, −0.02]	118.62 ***
Medium (25–49%)	18	5081	−0.10	[−0.16, −0.05]	−0.12	0.14	[−0.28, 0.04]	72.64 ***
Low (<25%)	21	7246	−0.15	[−0.19, −0.10]	−0.18	0.12	[−0.31, −0.04]	85.48 ***
**Contextual performance**								1.37
High (≥50%)	−	−	−	−	−	−	−	−
Medium (25–49%)	16	4646	−0.14	[−0.20, −0.08]	−0.17	0.14	[−0.33, −0.01]	66.96 ***
Low (<25%)	16	5341	−0.11	[−0.16, −0.07]	−0.15	0.13	[−0.29, −0.02]	55.31 ***
**Counterproductive work behavior**								0.00
High (≥50%)	−	−	−	−	−	−	−	−
Medium (25–49%)	8	2907	0.15	[0.07, 0.23]	0.17	0.14	[0.00, 0.34]	38.98 ***
Low (<25%)	8	2241	0.15	[0.05, 0.25]	0.18	0.16	[−0.01, 0.37]	46.83 ***
**Creativity**								31.49 ***
High (≥50%)	2	3340	−0.09	[−0.15, −0.03]	−0.10	0.05	[−0.15, −0.04]	6.80 **
Medium (25–49%)	2	933	0.12	[−0.16, 0.40]	0.14	0.25	[−0.17, 0.44]	38.33 ***
Low (<25%)	−	−	−	−	−	−	−	−
**Safety performance**								40.78 ***
High (≥50%)	3	2431	−0.20	[−0.21, −0.19]	−0.23	0.00	[−0.23, −0.23]	0.35
Medium (25–49%)	16	6600	−0.19	[−0.30, −0.08]	−0.23	0.27	[−0.57, 0.12]	364.38 ***
Low (<25%)	8	8843	−0.10	[−0.15, −0.05]	−0.12	0.09	[−0.22, −0.02]	48.49 ***

*Note*. *k* = number of samples; *N* = accumulated sample size; CI = confidence interval; $\bar{r}$*_o_* = sample-size weighted mean correlation; $\bar{r}$_c_ = correlation corrected for measurement error; *SD* = standard deviation; CV = credibility interval; *Q_B_* = homogeneity statistic *Q* between groups; *Q_W_* = homogeneity statistic *Q* within groups;—for this moderator level, the number of studies was too small (<2) to be included in the analysis. Union density rates: high (≥50%), medium (25–49%) and low (<25%). * *p* < 0.05, ** *p* < 0.01, *** *p* < 0.001.

## Supplementary Materials

A list of the studies included in the meta-analysis is available online at https://www.mdpi.com/1660-4601/16/14/2536/s1.

## Appendix A

**Classification of Countries into Macro-Level Indicators of Social Protection**

Table A1 provides the classification of countries into types of social welfare regimes (i.e., Scandinavian, Bismarckian, Southern European, Anglo-Saxon, Eastern European, and East Asian). Table A2 illustrates which countries were classified into low (<25%), medium (25−49%), and high (≥50%) union density.

**Table A1:** Classification of Countries by Type of Welfare Regime.

Scandinavian	Bismarckian	Southern European	Anglo-Saxon	Eastern European	East Asian
Denmark Finland Norway Sweden	Belgium France Germany Netherlands Switzerland	Italy Spain	Australia Canada Israel UK US	Czech Republic Hungary Poland Russia	South Korea Taiwan

Note: The typology is based on the classification of types of welfare regimes discussed by Kim et al. [24], based on Ferrera [106], Aspalter [108], and Lee & Ku [107].

**Table A2:** Classification of Countries by Union Density.

Low (<25%)	Medium (25−49%)	High (≥50%)
Brazil Cameroon Chile El Salvador Estonia France Guatemala Hungary Japan Korea Latvia Lithuania Malawi Mexico Netherlands Poland Romania Spain Switzerland Taiwan Tanzania United States	Australia Austria Canada China Croatia Czech Republic Germany Greece Ireland Israel Italy Kenya Luxembourg New Zealand Portugal Slovak Republic Slovenia South Africa Turkey United Kingdom	Belarus Belgium Cuba Cyprus Denmark Finland Iceland Norway Sweden

Note: The classification is based on statistics reported by the ILO [110] and the OECD [109], supplemented with information about union density in China [111] and Taiwan [112].

## References

[1] Burgard S.A., Brand J.E., House J.S. (2009). Perceived job insecurity and worker health in the United States. Soc. Sci. Med..

[2] Gallie D., Felstead A., Green F., Inanc H. (2017). The hidden face of job insecurity. Work Employ. Soc..

[3] De Witte H., De Cuyper N., Pienaar J. (2016). Review of 30 years of longitudinal studies on the association between job insecurity and health and well-being: Is there causal evidence?. Aust. Psychol..

[4] Jiang L., Lavaysse L.M. (2018). Cognitive and affective job insecurity: A meta-analysis and a primary study. J. Manag..

[5] Llosa J.A., Menéndez-Espina S., Agulló-Tomás E., Rodríguez-Suárez J. (2018). Job insecurity and mental health: A meta-analytical review of the consequences of precarious work in clinical disorders. An. Psicol..

[6] Shoss M.K. (2017). Job insecurity: An integrative review and agenda for future research. J. Manag..

[7] Flecker J., Fibich T., Kraemer K., Korunka C., Kubicek K. (2017). Socio-economic changes and the reorganization of work. Job Demands in a Changing World of Work.

[8] Lee C., Huang G.H., Ashford S.J., Morgeson F. (2018). Job insecurity and the changing workplace: Recent developments and the future trends in job insecurity research. Annual Review of Organizational Psychology and Organizational Behavior.

[9] Cheng G.H.L., Chan D.K.S. (2008). Who suffers more from job insecurity? A meta-analytic review. Appl. Psychol..

[10] Gilboa S., Shirom A., Fried Y., Cooper C. (2008). A meta-analysis of work demand stressors and job performance: Examining main and moderating effects. Pers. Psychol..

[11] Feather N.T., Rauter K.A. (2004). Organizational citizenship behaviours in relation to job status, job insecurity, organizational commitment and identification, job satisfaction and work values. J. Occup. Organ. Psychol..

[12] König C.J., Debus M.E., Häusler S., Lendenmann N., Kleinmann M. (2010). Examining occupational self-efficacy, work locus of control and communication as moderators of the job insecurity—Job performance relationship. Econ. Ind. Democr..

[13] Shoss M.K., Jiang L., Probst T.M. (2016). Bending without breaking: A two-study examination of employee resilience in the face of job insecurity. J. Occup. Health Psychol..

[14] Clarke S. (2012). The effect of challenge and hindrance stressors on safety behavior and safety outcomes: A meta-analysis. J. Occup. Health Psychol..

[15] Sverke M., Hellgren J. (2002). The nature of job insecurity: Understanding employment uncertainty on the brink of a new millennium. Appl. Psychol..

[16] Piccoli B., Reisel W.D., De Witte H. (2019). Understanding the relationship between job insecurity and performance: Hindrance or challenge effect?. J. Career Dev..

[17] Staufenbiel T., König C.J. (2010). A model for the effects of job insecurity on performance, turnover intention, and absenteeism. J. Occup. Organ. Psychol..

[18] Kim T.J., von dem Knesebeck O. (2015). Is an insecure job better for health than having no job at all? A systematic review of studies investigating the health-related risks of both job insecurity and unemployment. BMC Public Health.

[19] Sverke M., Hellgren J., Näswall K. (2002). No security: A meta-analysis and review of job insecurity and its consequences. J. Occup. Health Psychol..

[20] Heidemeier H., Moser K. (2009). Self–other agreement in job performance ratings: A meta-analytic test of a process model. J. Appl. Psychol..

[21] Podsakoff P.M., MacKenzie S.B., Podsakoff N.P. (2012). Sources of method bias in social science research and recommendations on how to control it. Annu. Rev. Psychol..

[22] Erlinghagen M. (2008). Self-perceived job insecurity and social context: A multi-level analysis of 17 European countries. Eur. Sociol. Rev..

[23] Berglund T., Vuori J., Blonk R., Price R.H. (2015). Flexicurity, job insecurity, and well-being in European labor markets. Sustainable Working Lives: Managing Work Transitions and Health throughout the Life Course.

[24] Kim I.-H., Muntaner C., Vahid F., Vives A., Vanroelen C., Benach J. (2012). Welfare states, flexible employment, and health: A critical review. Health Policy.

[25] Bender K.A., Sloane P.J. (1999). Trade union membership, tenure and the level of job insecurity. Appl. Econ..

[26] Sverke M., Hellgren J., Näswall K., Chirumbolo A., De Witte H., Goslinga S. (2004). Job Insecurity and Union Membership: European Unions in the Wake of Flexible Production.

[27] Greenhalgh L., Rosenblatt Z. (1984). Job insecurity: Toward conceptual clarity. Acad. Manag. Rev..

[28] Heaney C.A., Israel B.A., House J.S. (1994). Chronic job insecurity among automobile workers: Effects on job satisfaction and health. Soc. Sci. Med..

[29] De Cuyper N., Mäkikangas A., Kinnunen U., Mauno S., De Witte H. (2012). Cross-lagged associations between perceived external employability, job insecurity, and exhaustion: Testing gain and loss spirals according to the conservation of resources theory. J. Organ. Behav..

[30] De Beer L.T., Rothmann S., Pienaar J. (2016). Job insecurity, career opportunities, discrimination and turnover intention in post-apartheid South Africa: Examples of informative hypothesis testing. Int. J. Hum. Resour. Manag..

[31] Vander Elst T., De Witte H., De Cuyper N. (2014). The job insecurity scale: A psychometric evaluation across five European countries. Eur. J. Work Organ. Psychol..

[32] Keim A.C., Landis R.S., Pierce C.A., Earnest D.R. (2014). Why do employees worry about their jobs? A meta-analytic review of predictors of job insecurity. J. Occup. Health Psychol..

[33] Hellgren J., Sverke M., Isaksson K. (1999). A two-dimensional approach to job insecurity: Consequences for employee attitudes and well-being. Eur. J. Work Organ. Psychol..

[34] Huang G.-H., Lee C., Ashford S., Chen Z., Ren X. (2010). Affective job insecurity: A mediator of cognitive job insecurity and employee outcomes relationships. Int. Stud. Manag. Organ..

[35] Probst T.M. (2003). Development and validation of the Job Security Index and the Job Security Satisfaction Scale: A classical test theory and IRT approach. J. Occup. Organ. Psychol..

[36] Lazarus R.S., Folkman S. (1984). Stress, Appraisal, and Coping.

[37] Barling J., Kelloway E.K. (1996). Job insecurity and health: The moderating role of workplace control. Stress Med..

[38] Vander Elst T., Näswall K., Bernhard-Oettel C., De Witte H., Sverke M. (2016). The effect of job insecurity on employee health complaints: A within-person analysis of the explanatory role of threats to the manifest and latent benefits of work. J. Occup. Health Psychol..

[39] Jahoda M. (1981). Work, employment, and unemployment: Values, theories, and approaches in social research. Am. Psychol..

[40] Selenko E., Mäkikangas A., Stride C.B. (2017). Does job insecurity threaten who you are? Introducing a social identity perspective to explain well-being and performance consequences of job insecurity. J. Organ. Behav..

[41] Hobfoll S.E. (1989). Conservation of resources: A new attempt at conceptualizing stress. Am. Psychol..

[42] Rousseau D. (1995). Psychological Contract: Understanding of Written and Unwritten Agreements.

[43] De Cuyper N., De Witte H. (2007). Job insecurity in temporary versus permanent workers: Associations with attitudes, well-being, and behaviour. Work Stress.

[44] Piccoli B., De Witte H. (2015). Job insecurity and emotional exhaustion: Testing psychological contract breach versus distributive injustice as indicators of lack of reciprocity. Work Stress.

[45] Robinson S.L., Rousseau D.M. (1994). Violating the psychological contract: Not the exception but the norm. J. Organ. Behav..

[46] Richter A., Näswall K. (2018). Job insecurity and trust: Uncovering a mechanism linking job insecurity to well-being. Work Stress.

[47] Motowidlo S.J., Ilgen D., Klimoski R., Borman I. (2003). Job performance. Handbook of Psychology: Industrial and Organizational Psychology.

[48] Campbell J.P., Dunette M., Hough L.M. (1990). Modeling the performance prediction problem in industrial and organizational psychology. Handbook of Industrial and Organizational Psychology.

[49] Rotundo M., Sackett P.R. (2002). The relative importance of task, citizenship, and counterproductive performance to global ratings of job performance: A policy-capturing approach. J. Appl. Psychol..

[50] Viswesvaran C., Ones D.S. (2000). Perspectives on models of job performance. Int. J. Sel. Assess..

[51] Murphy K.R., Dillon R.F., Pellegrino J.W. (1989). Dimensions of job performance. Testing: Theoretical and Applied Perspectives.

[52] Hoffman B.J., Dilchert S., Schmitt N. (2012). A review of citizenship and counterproductive behaviors. Personnel Assessment and Selection.

[53] Organ D.W., Ryan K. (1995). A meta-analytic review of attitudinal and dispositional predictors of organizational citizenship behavior. Pers. Psychol..

[54] Sackett P., DeVore C., Anderson D.N., Ones D., Sinangil H., Viswesvaran C. (2001). Counterproductive behaviors at work. Handbook of Industrial, Work, & Organizational Psychology Volume 1: Personnel Psychology.

[55] Harari M.B., Reaves A.C., Viswesvaran C. (2016). Creative and innovative performance: A meta-analysis of relationships with task, citizenship, and counterproductive job performance dimensions. Eur. J. Work Organ. Psychol..

[56] Anderson N., Potočnik K., Zhou J. (2014). Innovation and creativity in organizations: A state-of-the-science review, prospective commentary, and guiding framework. J. Manag..

[57] Tierney P., Farmer S.M. (2002). Creative self-efficacy: Its potential antecedents and relationship to creative performance. Acad. Manag. J..

[58] Christian M.S., Bradley J.C., Wallace J.C., Burke M.J. (2009). Workplace safety: A meta-analysis of the roles of person and situation factors. J. Appl. Psychol..

[59] Beus J.M., Dhanani L.Y., McCord M.A. (2015). A meta-analysis of personality and workplace safety: Addressing unanswered questions. J. Appl. Psychol..

[60] Reisel W.D., Probst T.M., Swee-Lim C., Maloles C.M., König C.J. (2010). The effects of job insecurity on job satisfaction, organizational citizenship behavior, deviant behavior, and negative emotions of employees. Int. Stud. Manag. Organ..

[61] Shoss M.K., Probst T.M., Perrewé P.L., Halbesleben J.R.B., Rosen C.C. (2012). Multilevel outcomes of economic stress: An agenda for future research. The Role of the Economic Crisis on Occupational Stress and Well Being.

[62] Roll L.C., Siu O.-L., Li S.Y. (2015). The job insecurity-performance relationship in Germany and China: The buffering effect of uncertainty avoidance. Psihol. Resur. Um..

[63] Lee C., Bobko P., Chen Z.X. (2006). Investigation of the multidimensional model of job insecurity in China and the USA. Appl. Psychol..

[64] McKnight D.H., Phillips B., Hardgrave B.C. (2009). Which reduces IT turnover intention the most: Workplace characteristics or job characteristics?. Inf. Manag..

[65] De Cuyper N., Sulea C., Philippaers K., Fischmann G., Iliescu D., De Witte H. (2014). Perceived employability and performance: Moderation by felt job insecurity. Pers. Rev..

[66] Lin X., Leung K. (2014). What signals does procedural justice climate convey? The roles of group status, and organizational benevolence and integrity. J. Organ. Behav..

[67] Huang G.H., Wellman N., Ashford S.J., Lee C., Wang L. (2017). Deviance and exit: The organizational costs of job insecurity and moral disengagement. J. Appl. Psychol..

[68] Lim V.K.G. (1996). Job insecurity and its outcomes: Moderating effects of work-based and nonwork-based social support. Hum. Relat..

[69] Probst T.M., Stewart S.M., Gruys M.L., Tierney B.W. (2007). Productivity, counterproductivity and creativity: The ups and downs of job insecurity. J. Occup. Organ. Psychol..

[70] Conway N., Kiefer T., Hartley J., Briner R.B. (2014). Doing more with less? Employee reactions to psychological contract breach via target similarity or spillover during public sector organizational change. Br. J. Manag..

[71] Choi S.B., Cundiff N., Kim K., Akhatib S.N. (2018). The effect of work–family conflict and job insecurity on innovative behaviour of Korean workers: The mediating role of organisational commitment and job satisfaction. Int. J. Innov. Manag..

[72] Punnett B.J., Greenidge D., Ramsey J. (2007). Job attitudes and absenteeism: A study in the English speaking Caribbean. J. World Bus..

[73] Teng E., Zhang L., Qiu Y. (2018). Always bad for creativity? An affect-based model of job insecurity and the moderating effects of giving support and receiving support. Econ. Ind. Democr..

[74] Vander Elst T., De Cuyper N., Baillien E., Niesen W., De Witte H. (2016). Perceived control and psychological contract breach as explanations of the relationships between job insecurity, job strain and coping reactions: Towards a theoretical integration. Stress Health J. Int. Soc. Investig. Stress.

[75] Jiang L., Probst T.M. (2016). A multilevel examination of affective job insecurity climate on safety outcomes. J. Occup. Health Psychol..

[76] Wang H., Liu X., Luo H., Ma B., Liu S. (2016). Linking procedural justice with employees’ work outcomes in China: The mediating role of job security. Soc. Indic. Res..

[77] Probst T.M., Petitta L., Barbaranelli C., Lavaysse L.M. (2016). Moderating effects of contingent work on the relationship between job insecurity and employee safety. Saf. Sci..

[78] Hunter J.E., Schmidt F.L. (2017). Methods of Meta-Analysis: Correcting Error and Bias in Research Findings.

[79] De Witte H., Vander Elst T., De Cuyper N., Vuori J., Blonk R., Price R.H. (2015). Job insecurity, health and well-being. Sustainable Working Lives: Managing Work Transitions and Health throughout the Life Course.

[80] Hur H. (2019). Job security matters: A systematic review and meta-analysis of the relationship between job security and work attitudes. J. Manag. Organ..

[81] Zapf D., Dormann C., Frese M. (1996). Longitudinal studies in organizational stress research: A review of the literature with reference to methodological issues. J. Occup. Health Psychol..

[82] Bollen K.A. (1989). Structural Equations with Latent Variables.

[83] Armstrong-Stassen M. (2002). Designated redundant but escaping lay-off: A special group of lay-off survivors. J. Occup. Organ. Psychol..

[84] Wang H.J., Lu C.Q., Siu O.L. (2015). Job insecurity and job performance: The moderating role of organizational justice and the mediating role of work engagement. J. Appl. Psychol..

[85] Chan D., Vandenberg R.J., Lance C.E. (2009). So why ask me? Are self-report data really that bad?. Statistical and Methodological Myths and Urban Legends: Doctrine, Verity and Fable in the Organizational and Social Sciences.

[86] Podsakoff P.M., MacKenzie S.B., Lee J.-Y., Podsakoff N.P. (2003). Common method biases in behavioral research: A critical review of the literature and recommended remedies. J. Appl. Psychol..

[87] Donaldson S.I., Grant-Vallone E.J. (2002). Understanding self-report bias in organizational behavior research. J. Bus. Psychol..

[88] De Cuyper N., Schreurs B., Vander Elst T., Baillien E., De Witte H. (2014). Exemplification and perceived job insecurity: Associations with self-rated performance and emotional exhaustion. J. Pers. Psychol..

[89] Probst T.M., Jiang L. (2017). European flexicurity policies: Multilevel effects on employee psychosocial reactions to job insecurity. Saf. Sci..

[90] Sender A., Staffelbach B., Arnold A. (2016). Job security as a threatened resource: Reactions to job insecurity in culturally distinct regions. Int. J. Hum. Resour. Manag..

[91] Berglund T., Furåker B., Vulkan P. (2014). Is job insecurity compensated for by employment and income security?. Econ. Ind. Democr..

[92] Probst T.M., Lawler J. (2006). Cultural values as moderators of employee reactions to job insecurity the role of individualism and collectivism. Appl. Psychol. Int. Rev..

[93] Anderson C.J., Pontusson J. (2007). Workers, worries and welfare states: Social protection and job insecurity in 15 OECD countries. Eur. J. Political Res..

[94] Hipp L. (2016). Insecure times? Workers’ perceived job and labor market security in 23 OECD countries. Soc. Sci. Res..

[95] Ding H. (2014). Unemployment and welfare state: What do the data tell us?. J. Soc. Sci. Policy Implic..

[96] Marx P. (2014). The effect of job insecurity and employability on preferences for redistribution in Western Europe. J. Eur. Soc. Policy.

[97] Visser J. (2002). Why fewer workers join unions in Europe: A social custom explanation of membership trends. Br. J. Ind. Relat..

[98] Näswall K., Sverke M., Day A., Kelloway E.K., Hurrell J. (2014). Unions and changes in working life: New challenges, new opportunities. Workplace Well-Being: How to Build Psychologically Healthy Workplaces.

[99] Sverke M., Hellgren J. (2001). Exit, voice and loyalty reactions to job insecurity in Sweden: Do unionized and non-unionized employees differ?. Br. J. Ind. Relat..

[100] Näswall K., Hellgren J., Sverke M., De Witte H. (2017). We get by with a little help from our unions: Psychological contract violations and downsizing. Job Insecurity, Union Involvement and Union Activism.

[101] Goslinga S., Hellgren J., Chirumbolo A., De Witte H., Näswall K., Sverke M. (2005). The role of union support in coping with job insecurity: A study among union members from three European countries. SA J. Ind. Psychol..

[102] Barling J., Fullagar C., Kelloway K.K. (1992). The Union and Its Members: A Psychological Approach.

[103] Ferrie J.E. (2001). Is job insecurity harmful to health?. J. R. Soc. Med..

[104] Probst T.M., Jiang L., Benson W., Klehe U.-C., Hooft E.V. (2018). Job insecurity and anticipated job loss: A primer and exploration of possible interventions. The Oxford Handbook of Job Loss and Job Search.

[105] De Witte H. (2005). Job insecurity: Review of the international literature on definitions, prevalence, antecedents and consequences. SA J. Ind. Psychol..

[106] Ferrera M. (1996). The ‘southern model’ of welfare in social Europe. J. Eur. Soc. Policy.

[107] Lee Y.J., Ku Y.W. (2007). East Asian welfare regimes: Testing the hypothesis of the developmental welfare state. Soc. Policy Adm..

[108] Aspalter C. (2006). The East Asian welfare model. Int. J. Soc. Welf..

[109] The Organisation for Economic Co-operation and Development (OECD) (2018). Trade Union Density.

[110] The International Labour Organization (ILO) (2018). Trade Union Density (%).

[111] Traub-Merz R. (2011). All China Federation of Trade Unions: Structure, Functions and the Challenge of Collective Bargaining.

[112] Lee Y. (2011). Militants or Partisans: Labor Unions and Democratic Politics in Korea and Taiwan.

[113] Whitener E.M. (1990). Confusion of confidence intervals and credibility intervals in meta-analysis. J. Appl. Psychol..

[114] Hedges L.V., Olkin I. (1985). Statistical Methods for Meta-Analysis.

[115] Mathieu J.E., Zajac D.M. (1990). A review and meta-analysis of the antecedents, correlates, and consequences of organizational commitment. Psychol. Bull..

[116] De Spiegelaere S., Van Gyes G., De Witte H., Niesen W., Van Hootegem G. (2014). On the relation of job insecurity, job autonomy, innovative work behaviour and the mediating effect of work engagement. Creat. Innov. Manag..

[117] Van Hootegem A., Niesen W., De Witte H. (2019). Does job insecurity hinder innovative work behaviour? A threat rigidity perspective. Creat. Innov. Manag..

[118] Staw B.M., Sandelands L.E., Dutton J.E. (1981). Threat-rigidity effects in organizational behavior: A multilevel analysis. Adm. Sci. Q..

[119] Karatepe O.M., Vatankhah S. (2014). The effects of high-performance work practices and job embeddedness on flight attendants’ performance outcomes. J. Air Transp. Manag..

[120] Probst T.M., Gailey N.J., Jiang L., Bohle S.L. (2017). Psychological capital: Buffering the longitudinal curvilinear effects of job insecurity on performance. Saf. Sci..

[121] Selenko E., Mäkikangas A., Mauno S., Kinnunen U. (2013). How does job insecurity relate to self-reported job performance? Analysing curvilinear associations in a longitudinal sample. J. Occup. Organ. Psychol..

[122] Fischmann G., Sulea C., Kovacs P., Iliescu D., De Witte H. (2015). Qualitative and quantitative job insecurity: Relations with nine types of performance. Psihol. Resur. Um..

[123] Sverke M., De Witte H., Näswall K., Hellgren J. (2010). European perspectives on job insecurity: Editorial introduction. Econ. Ind. Democr..

[124] Pitariu A.H., Ployhart R.E. (2010). Explaining change: Theorizing and testing dynamic mediated longitudinal relationships. J. Manag..

[125] Podsakoff N.P., Whiting S.W., Podsakoff P.M., Blume B.D. (2009). Individual-and organizational-level consequences of organizational citizenship behaviors: A meta-analysis. J. Appl. Psychol..

[126] Bambra C. (2007). Going beyond the three worlds of welfare capitalism: Regime theory and public health research. J. Epidemiol. Community Health.

[127] Jang S., Shen W., Allen T.D., Zhang H.Y. (2018). Societal individualism–collectivism and uncertainty avoidance as cultural moderators of relationships between job resources and strain. J. Organ. Behav..

[128] König C.J., Probst T.M., Staffen S., Graso M. (2011). A Swiss–US comparison of the correlates of job insecurity. Appl. Psychol..

[129] Debus M.E., Probst T.M., König C.J., Kleinmann M. (2012). Catch me if I fall! Enacted uncertainty avoidance and the social safety net as country-level moderators in the job insecurity–job attitudes link. J. Appl. Psychol..

[130] Mauno S., Leskinen E., Kinnunen U. (2001). Multi-wave, multi-variable models of job insecurity: Applying different scales in studying the stability of job insecurity. J. Organ. Behav. Int. J. Ind. Occup. Organ. Psychol. Behav..

[131] De Witte H., De Cuyper N., Handaja Y., Sverke M., Näswall K., Hellgren J. (2010). Associations between quantitative and qualitative job insecurity and well-being. Int. Stud. Manag. Organ..

[132] Schreurs B., Guenter H., Jawahar I.M.J., De Cuyper N. (2015). Speaking up when feeling job insecure: The moderating role of punishment and reward sensitivity. J. Organ. Chang. Manag..

[133] Hellgren J., Sverke M. (2003). Does job insecurity lead to impaired well-being or vice versa? Estimation of cross-lagged effects using latent variable modelling. J. Organ. Behav..

[134] Frese M., Zapf D., Cooper C.L., Payne R. (1988). Methodological issues in the study of work stress: Objective vs. subjective measurement of work stress and the question of longitudinal studies. Causes, Coping and Consequences of Stress at Work.

[135] Selig J.P., Preacher K.J., Little T.D. (2012). Modeling time-dependent association in longitudinal data: A lag as moderator approach. Multivar. Behav. Res..

[136] Klug K., Bernhard-Oettel C., Mäkikangas A., Kinnunen U., Sverke M. (2019). Development of perceived job insecurity among young workers: A latent class growth analysis. Int. Arch. Occup. Environ. Health.

[137] Kinnunen U., Mäkikangas A., Mauno S., De Cuyper N., De Witte H. (2014). Development of perceived job insecurity across two years: Associations with antecedents and employee outcomes. J. Occup. Health Psychol..

[138] Klug K., Bernhard-Oettel C., Selenko E., Sverke M., Griep Y., Hansen S., Hofmans J., Vantilborgh T. A dynamic perspective on job insecurity: Temporal and person-oriented perspectives. Handbook of Dynamic Organizational Behavior.

[139] Lance C.E., Dawson B., Birkelbach D., Hoffman B.J. (2010). Method effects, measurement error, and substantive conclusions. Organ. Res. Methods.

[140] Dalton D.R., Aguinis H., Dalton C.M., Bosco F.A., Pierce C.A. (2012). Revisiting the file drawer problem in meta-analysis: An assessment of published and nonpublished correlation matrices. Pers. Psychol..

[141] DeNisi A.S., Murphy K.R. (2017). Performance appraisal and performance management: 100 years of progress?. J. Appl. Psychol..

[142] Freeman R.B., Medoff J.L. (1984). What Do Unions Do?.

[143] Hellgren J., Sverke M. (2001). Unionized employees’ perceptions of role stress and fairness during organizational downsizing: Consequences for job satisfaction, union satisfaction and well-being. Econ. Ind. Democr..

