# Supplementary material for: A Meta-Analysis of Job Insecurity and Employee Performance: Testing Temporal Aspects, Rating Source, Welfare Regime, and Union Density as Moderators
Source: Int J Environ Res Public Health. 2019 Jul 16;16(14):2536. doi: 10.3390/ijerph16142536 (PMC6678210; doi:10.3390/ijerph16142536)
Supplement: Supplementary file 1 [file ijerph-16-02536-s001.pdf]

Supplementary Material

Studies Included in the Meta-analysis

Abiodun, A.J.; Osibanjo, O.A.; Adeniji, A.A.; Iyere-Okojie, E. Modelling the relationship between job demands, work attitudes and performance among nurses in a transition economy. *International Journal of Healthcare Management* **2014**, *7*, 257-264, doi:10.1179/2047971914Y.0000000073.

Akanni, A.A.; Oladejo, O.E.; Oduaran, C.A. Work-life balance, job insecurity and counterproductive work behaviour among brewery workers. *North American Journal of Psychology* **2018**, *20*, 289-300.

Akgunduz, Y.; Eryilmaz, G. Does turnover intention mediate the effects of job insecurity and co-worker support on social loafing? *International Journal of Hospitality Management* **2018**, *68*, 41-49, doi:10.1016/j.ijhm.2017.09.010.

Armstrong-Stassen, M. Production workers reactions to a plant closing: The role of transfer, stress and support. *Anxiety, Stress and Coping: An International Journal* **1993**, *6*, 201-214, doi:10.1080/10615809308248380.

Armstrong-Stassen, M. Coping with transition: A study of layoff survivors. *Journal of Organizational Behavior* **1994**, *15*, 597-621, doi:10.1002/job.4030150705.

Armstrong-Stassen, M. Designated redundant but escaping lay-off: A special group of lay-off survivors. *Journal of Occupational and Organizational Psychology* **2002**, 1-13, doi:10.1348/096317902167603.

Armstrong-Stassen, M. Job transfer during organizational downsizing: a comparison of promotion and lateral transfers. *Group & Organization Management* **2003**, *28*, 392-415, doi:10.1177/1059601102250824.

Armstrong-Stassen, M., Wagar, T. H., Cattaneo, R. J. Work-group membership (in)stability and survivors' reactions to organizational downsizing. *Journal of Applied Social Psychology* **2004**, *34*, 2023-2044, doi:10.1111/j.1559-1816.2004.tb02689.x.

Baran, M.; Kanten, P.; Kanten, S.; Yaşlıoğlu, M. An empirical research on the relationship between job insecurity and employee health & safety. *Ege Academic Review* **2009**, *9*, 969-976, doi:10.21121/eab.2009319698.

Bartol, K.M.; Liu, W.; Zeng, X.; Wu, K. Social exchange and knowledge sharing among knowledge workers: The moderating role of perceived job security. *Management and Organization Review* **2009**, *5*, 223-240, doi:10.1111/j.1740-8784.2009.00146.x.

Bohle, S.A.L.; Alonso, A.R.M. The effect of procedural fairness and supervisor support in the relationship between job insecurity and organizational citizenship behavior. *RBGN-Rev. Bras. Gest. Negocios* **2017**, *19*, 337-357, doi:10.7819/rbgn.v0i0.3023.

Bouzari, M.; Karatepe, O.M. Antecedents and outcomes of job insecurity among salespeople. *Mark. Intell. Plan.* **2018**, *36*, 290-302, doi:10.1108/mip-11-2017-0314.

Brandes, P.; Castro, S.L.; James, M.S.; Martinez, A.D.; Matherly, T.A.; Ferris, G.R.; Hochwarter, W.A. The interactive effects of job insecurity and organizational cynicism on work effort following a layoff. *Journal of Leadership & Organizational Studies* **2008**, *14*, 233-247, doi:10.1177%2F1071791907311967.

Bronkhorst, B. Behaving safely under pressure: The effects of job demands, resources, and safety climate on employee physical and psychosocial safety behavior. *Journal of Safety Research* **2015**, *55*, 63-72, doi:http://dx.doi.org/10.1016/j.jsr.2015.09.002.

Chiamaka, O.; Oguegbe, T.M.; Aguanunu, R. Exploratory study of job insecurity and entrepreneurial intention as correlates of counterproductive work behaviour. *International Journal of Academic Research in Business and Social Sciences* **2014**, *4*, 41-52, doi:10.6007/IJARBSS/v4-i5/822.

Chirumbolo, A. The impact of job insecurity on counterproductive work behaviors: The moderating role of honesty–humility personality trait. *The Journal of Psychology* **2014**, 1-16, doi:10.1080/00223980.2014.916250.

Chirumbolo, A.; Areni, A. Job insecurity influence on job performance and mental health: Testing the moderating effect of the need for closure. *Economic and Industrial Democracy* **2010**, *31*, 195-214, doi:10.1177/0143831x09358368.

Chiu, S.-F.; Lin, S.-T.; Han, T.-S. Employment status and employee service-oriented organizational citizenship behaviour: The mediating roles of internal mobility opportunity and job insecurity. *The Career Development International* **2015**, *20*, 133-146, doi:http://dx.doi.org/10.1108/CDI-07-2014-0096.

Choi, S.B.; Cundiff, N.; Kim, K.; Akhatib, S.N. The effect of work-family conflict and job insecurity on innovative behaviour of Korean workers: The mediating role of organisational commitment and job satisfaction. *Int. J. Innov. Manag.* **2018**, *22*, 29, doi:10.1142/s1363919618500032.

Chung, G.H.; Du, J.; Choi, J.N. How do employees adapt to organizational change driven by cross-border M&As? A case in China. *Journal of World Business* **2014**, *49*, 78-86, doi:10.1016/j.jwb.2013.01.001.

Clinton, M.; Bernhard-Oettel, C.; Rigotti, T.; Jong, J.d. Expanding the temporal context of research on non-permanent work: Previous experience, duration of and time remaining on contracts and employment continuity expectations. *Career Development International* **2011**, *16*, 114-139, doi:10.1108/13620431111115596.

Colarelli, S.M.; Dean, R.A.; Konstans, C. Comparative effects of personal and situational influences on job outcomes of new professionals. *Journal of Applied Psychology* **1987**, *72*, 558-566.

Conway, N.; Kiefer, T.; Hartley, J.; Briner, R.B. Doing more with less? Employee reactions to psychological contract breach via target similarity or spillover during public sector organizational change. *British Journal of Management* **2014**, *25*, 737-754, doi:10.1111/1467-8551.12041.

Costa, S.; Neves, P. Job insecurity and work outcomes: The role of psychological contract breach and positive psychological capital. *Work & Stress* **2017**, *31*, 375-394, doi:http://dx.doi.org/10.1080/02678373.2017.1330781.

De Cuyper, N.; De Witte, H. Job insecurity: Mediator or moderator of the relationship between type of contract and various outcomes. *SA Journal of Industrial Psychology* **2005**, *31*, 79-86, doi:10.4102/sajip.v31i4.211.

De Cuyper, N., & De Witte, H. The impact of job insecurity and contract type on attitudes, well-being and behavioural reports: a psychological contract perspective. *Journal of Occupational and Organizational Psychology* **2006**, *79*, 395-409, doi:10.1348/096317905X53660.

De Cuyper, N.; De Witte, H. Job insecurity in temporary versus permanent workers: Associations with attitudes, well-being, and behaviour. *Work & Stress* **2007**, *21*, 65-84, doi:10.1080/02678370701229050.

De Cuyper, N.; Schreurs, B.; Vander Elst, T.; Baillien, E.; De Witte, H. Exemplification and perceived job insecurity: Associations with self-rated performance and emotional exhaustion. *Journal of Personnel Psychology* **2014**, *13*, 1, doi:10.1027/1866-5888/a000099.

De Cuyper, N.; Sulea, C.; Philippaers, K.; Fischmann, G.; Iliescu, D.; De Witte, H. Perceived employability and performance: Moderation by felt job insecurity. *Personnel Review* **2014**, *43*, 536-552, doi:10.1108/pr-03-2013-0050.

De Spiegelaere, S.; Van Gyes, G.; De Witte, H.; Niesen, W.; Van Hootegem, G. On the relation of job insecurity, job autonomy, innovative work behaviour and the mediating effect of work engagement. *Creativity & Innovation Management* **2014**, *23*, 318-330, doi:10.1111/caim.12079.

Dubinsky, A.J.; Kotabe, M.; Lim, C.U.; Wagner, W. The impact of values on salespeople´s job responses: A cross-national investigation. *Journal of Business Research* **1997**, *39*, 195-208, doi:10.1016/S0148-2963(96)00204-4.

Emberland, J.S.; Rundmo, T. Implications of job insecurity perceptions and job insecurity responses for psychological well-being, turnover intentions and reported risk behavior. *Safety Science* **2010**, *48*, 452-459, doi:10.1016/j.ssci.2009.12.002.

Feather, N.T.R.; Rauter, K.A. Organizational citizenship behaviours in relation to job status, job insecurity, organizational commitment and identification, job satisfaction and work values. *Journal of Occupational and Organizational Psychology* **2004**, *77*, 81-94, doi:10.1348/096317904322915928.

Fields, D.; Dingman, M.E.; Roman, P.M.; Blum, T.C. Exploring predictors of alternative job changes. *Journal of Occupational and Organizational Psychology* **2005**, *78*, 63-82, doi:10.1348/096317904X22719.

Filipkowski, M.; Johnson, C.M. Comparisons of performance and job insecurity in union and nonunion sites of a manufacturing company. *Journal of Organizational Behavior Management* **2008**, *28*, 218-237, doi:10.1080/01608060802454437.

Fischmann, G.; Sulea, C.; Kovacs, P.; Iliescu, D.; De Witte, H. Qualitative and quantitative job insecurity: relations with nine types of performance. *Psihologia Resurselor Umane* **2015**, *13*, 152-164.

Fried, Y.; Slowik, L.H.; Shperling, Z.; Franz, C.; Ben-David, H.a.; Avital, N.; Yeverechyahu, U. The moderating effect of job security on the relation between role clarity and job performance: A longitudinal field study. *Human Relations* **2003**, *56*, 787-805, doi:10.1177/00187267030567002.

Huang, G.H.; Wellman, N.; Ashford, S.J.; Lee, C.; Wang, L. Deviance and exit: The organizational costs of job insecurity and moral disengagement. *Journal of Applied Psychology* **2017**, *102*, 26-42, doi:10.1037/apl0000158.

Huang, G.-h.; Niu, X.; Lee, C.; Ashford, S.J. Differentiating cognitive and affective job insecurity: Antecedents and outcomes. *Journal of Organizational Behavior* **2012**, *33*, 752-769, doi:10.1002/job.1815.

Hui, C.; Lee, C. Moderating effects of organization-based self-esteem on organizational uncertainty: Employee response relationships. **2000**, *56*, 215-232, doi:10.1016/S0149-2063(99)00043-4.

Idrees, M.D.; Hafeez, M.; Kim, J.Y. Workers' age and the impact of psychological factors on the perception of safety at construction sites. *Sustainability* **2017**, *9*, 15, doi:10.3390/su9050745.

Jacobs, M.; Pienaar, J. Stress, coping and safety compliance in a multinational gold mining company. *International Journal of Occupational Safety and Ergonomics* **2017**, *23*, 152-161, doi:10.1080/10803548.2016.1263476.

Jiang, L.; Probst, T.M. A multilevel examination of affective job insecurity climate on safety outcomes. *Journal of occupational health psychology* **2016**, *21*, 366-377, doi:10.1037/0021-9010.75.6.698 http://dx.doi.org/10.1037/ocp0000014.

Johnson, C.D.; Messe, L.A.; Crano, W.D. Predicting job performance of low income workers: The work opinion questionnaire. *Personnel Psychology* **1984**, *37*, 291-299, doi:10.1111/j.1744-6570.1984.tb01451.x.

Judeh, M. Examining the relationship between organizational justice, job security, and organizational citizenship behavior in the Jordanian banks: A structural equation modeling perspective. *Jordan Journal of Business Administration* **2012**, *153*, 1-44.

Kang, D.-s.; Gold, J.; Kim, D. Responses to job insecurity: The impact on discretionary extra-role and impression management behaviors and the moderating role of employability. *Career Development International* **2012**, *17*, 314-332, doi:10.1108/13620431211255815.

Karatepe, O.M.; Vatankhah, S. The effects of high-performance work practices and job embeddedness on flight attendants' performance outcomes. *Journal of Air Transport Management* **2014**, *37*, 27-35, doi:10.1016/j.jairtraman.2014.01.008.

King, J.E. White-collar reactions to job insecurity and the role of the psychological contract: Implications for human resource management. *Human Resource Management* **2000**, *39*, 79-92, doi:10.1002/(SICI)1099-050X(200021)39:1%3C79::AID-HRM7%3E3.0.CO;2-A.

Kraimer, M.L.; Wayne, S.J.; Liden, R.C.; Sparrowe, R.T. The role of job security in understanding the relationship between employees' perceptions of temporary workers and employees' performance. **2005**, *90*, 389-398, doi:10.1037/0021-9010.90.2.389.

König, C.J.; Debus, M.E.; Haüsler, S.; Lendenmann, N.; Kleinmann, M. Examining occupational self-efficacy, work locus of control and communication as moderators of the job insecurity--job performance relationship. *Economic and Industrial Democracy* **2010**, *31*, 231-247, doi:10.1177/0143831x09358629.

Lam, C.F.; Liang, J.; Ashford, S.J.; Lee, C. Job insecurity and organizational citizenship behavior: Exploring curvilinear and moderated relationships. *Journal of Applied Psychology* **2015**, *100*, 499-510, doi:10.1037/a0038659.

Lawrence, E.R.; Kacmar, K.M. Exploring the impact of job insecurity on employees' unethical behavior. *Bus. Ethics Q.* **2017**, *27*, 39-70, doi:10.1017/beq.2016.58.

Lee, C.; Bobko, P.; Chen, Z.X. Investigation of the multidimensional model of job insecurity in China and the USA. *Applied Psychology* **2006**, *55*, 512-540, doi:10.1111/j.1464-0597.2006.00233.x.

Lim, V.K.G. Job insecurity and its outcomes: Moderating effects of work-based and nonwork-based social support. *Human Relations* **1996**, *49*, 171-194, doi:10.1177/001872679604900203.

Lin, X.; Leung, K. What signals does procedural justice climate convey? The roles of group status, and organizational benevolence and integrity. *Journal of Organizational Behavior* **2014**, *35*, 464-488, doi:10.1002/job.1899.

López Bohle, S.; Bal, P.M.; Jansen, P.G.W.; Leiva, P.I.; Alonso, A.M. How mass layoffs are related to lower job performance and OCB among surviving employees in Chile: An investigation of the essential role of psychological contract. *International Journal of Human Resource Management* **2016**, 10.1080/09585192.2016.1138988, 1-24, doi:10.1080/09585192.2016.1138988.

Låstad, L.; Näswall, K.; Berntson, E.; Seddigh, A.; Sverke, M. The roles of shared perceptions of individual job insecurity and job insecurity climate for work- and health-related outcomes: A multilevel approach. *Economic and Industrial Democracy* **2016**, 10.1177/0143831x16637129, doi:10.1177/0143831x16637129.

Mahmoud, A.B.; Reisel, W.D. Exploring personal experience of wartime crisis effects on job insecurity in Syria. *Psihologia Resurselor Umane* **2015**, *13*, 245-256.

Masia, U.; Pienaar, J. Unravelling safety compliance in the mining industry: examining the role of work stress, job insecurity, satisfaction and commitment as antecedents. *SA Journal of Industrial Psychology* **2011**, *37*, 01-10, doi:10.1402/sajip.v37i1.937.

Mathies, C.; Ngo, L.V. New insights into the climate–attitudes–outcome framework: Empirical evidence from the Australian service sector. *Australian Journal of Management* **2014**, *39*, 473-491, doi:10.1177/0312896213495054.

McKnight, D.H.; Phillips, B.; Hardgrave, B.C. Which reduces IT turnover intention the most: Workplace characteristics or job characteristics? *Information & Management* **2009**, *46*, 167-174, doi:10.1016/j.im.2009.01.002

Noble, C.H. The influence of job security on field sales manager satisfaction: Exploring frontline tensions. *Journal of Personal Selling and Sales Management* **2008**, *28*, 247-262, doi:10.2753/pss0885-3134280303.

Parker, S.K.; Axtell, C.M.; Turner, N. Designing a safer workplace: Importance of job autonomy, communication quality, and supportive supervisors. *Journal of Occupational Health Psychology* **2001**, *6*, 211-228, doi:10.1037//1076-8998.6.3.211.

Pearce, J.L.; Branzyicki, I.; Bakasci, G. Person-based reward systems: A theory of organizational reward practices in reform-communist organizations. *Journal of Organizational Behavior* **1994**, *15*, 261-282, doi:10.1002/job.4030150307

Piccoli, B.; De Witte, H.; Reisel, W.D. Job insecurity and discretionary behaviors: Social exchange perspective versus group value model. *Scandinavian journal of psychology* **2017**, *58*, 69-79, doi:10.1111/sjop.12340.

Probst, T.M. Wedded to the job: Moderating effects of job involvement on the consequences of job insecurity. *Journal of Occupational Health Psychology* **2000**, *5*, 63-73, doi:10.1037/1076-8998.5.1.63.

Probst, T.M. Safety and insecurity: Exploring the moderating effect of organizational safety climate. *Journal of occupational health psychology* **2004**, *9*, 3-10, doi:10.1037/1076-8998.9.1.3.

Probst, T.M. Countering the negative effects of job insecurity through participative decision making: lessons from the demand-control model. *Journal of Occupational Health Psychology* **2005**, *10*, 320-329, doi:10.1037/1076-8998.10.4.320.

Probst, T.M.; Brubaker, T.L. The effects of job insecurity on employee safety outcomes: Cross-sectional and longitudinal explorations. *Journal of Occupational Health Psychology* **2001**, *6*, 139-159, doi:10.1037//1076-8998.6.2.139.

Probst, T.M.; Ekore, J.O. An exploratory study of the costs of job insecurity in Nigeria. *International Studies of Management & Organization* **2010**, *40*, 92-104, doi:10.2753/IMO0020-8825400106.

Probst, T.M.; Gailey, N.J.; Jiang, L.; Bohle, S.L. Psychological capital: Buffering the longitudinal curvilinear effects of job insecurity on performance. *Safety Science* **2017**, *100*, 74-82, doi:10.1016/j.ssci.2017.02.002.

Probst, T.M.; Lawler, J. Cultural values as moderators of employee reactions to job insecurity the role of individualism and collectivism. *Applied Psychology: An International Review* **2006**, *55*, 234-254, doi:10.1111/j.1464-0597.2006.00239.x.

Probst, T.M.; Petitta, L.; Barbaranelli, C. Comparing recall vs. recognition measures of accident under-reporting: A two-country examination. *Accident Analysis and Prevention* **2017**, *106*, 1-9, doi:http://dx.doi.org/10.1016/j.aap.2017.05.006.

Probst, T.M.; Petitta, L.; Barbaranelli, C.; Lavaysse, L.M. Moderating effects of contingent work on the relationship between job insecurity and employee safety. *Safety Science* **2016**, http://dx.doi.org/10.1016/j.ssci.2016.08.008, doi:http://dx.doi.org/10.1016/j.ssci.2016.08.008.

Probst, T.M.; Stewart, S.M.; Gruys, M.L.; Tierney, B.W. Productivity, counterproductivity and creativity: The ups and downs of job insecurity. *Journal of Occupational and Organizational Psychology* **2007**, *80*, 479-497, doi:10.1348/096317906x159103.

Punnett, B.J.; Greenidge, D.; Ramsey, J. Job attitudes and absenteeism: A study in the English speaking Caribbean. *Journal of World Business* **2007**, *42*, 214-227, doi:10.1016/j.jwb.2007.02.006.

Reisel, W.D., Probst, T. M., Swee-Lim, C., Maloles, C. M., & König, C. J. The effects of job insecurity on job satisfaction, organizational citizenship behavior, deviant behavior, and negative emotions of employees. *International Studies of Management & Organization* **2010**, *40*, 74-91, doi:10.2753/IMO0020-8825400105.

Roll, L.C.; Siu, O.-l.; Li, S.Y. The job insecurity-performance relationship in Germany and China: The buffering effect of uncertainty avoidance. *Psihologia Resurselor Umane* **2015**, *13*, 165-178.

Ruvio, A.R., Z. Job insecurity among Israeli schoolteachers: Sectoral profiles and organizational implications. *Journal of Educational Administration* **1998**, *37*, 139-158, doi:10.1108/09578239910263024.

Schreurs, B. H., van Emmerik, IJ H., Günter, H., & Germeys, F. A weekly diary study on the buffering role of social support in the relationship between job insecurity and employee performance. *Human Resource Management* **2012**, *51*, 259-279, doi:10.1002/hrm.21465.

Selenko, E.; Mäkikangas, A.; Stride, C.B. Does job insecurity threaten who you are? Introducing a social identity perspective to explain well-being and performance consequences of job insecurity. *Journal of Organizational Behavior* **2017**, *38*, 856-875, doi:10.1002/job.2172.

Selenko, E.; Mäkikangas, A.; Mauno, S.; Kinnunen, U. How does job insecurity relate to self-reported job performance? Analysing curvilinear associations in a longitudinal sample. *Journal of Occupational and Organizational Psychology* **2013**, *86*, 522-542, doi:10.1111/joop.12020.

Shoss, M.K.; Jiang, L.; Probst, T.M. Bending without breaking: A two-study examination of employee resilience in the face of job insecurity. *Journal of Occupational Health Psychology* **2016**, *23*, 112-126, doi:10.1037/ocp0000060.

Sora, B.M.; González-Morales, M.G.; Caballer, A.; Peiró, J.M. Consequences of job insecurity and the moderator role of occupational group. *The Spanish Journal of Psychology* **2011**, *14*, 820-831, doi:10.5209/rev_SJOP.2011.v14.n2.29.

Staufenbiel, T.; König, C.J. A model for the effects of job insecurity on performance, turnover intention, and absenteeism. *Journal of Occupational and Organizational Psychology* **2010**, *83*, 101-117, doi:10.1348/096317908x401912.

Stepina, L.P.; Perrewe, P.L. The stability of comparative referent choice and feelings of inequity: A longitudinal field study. *Journal of Organizational Behavior* **1991**, *12*, 185-200, doi:10.1002/job.4030120303.

Størseth, F. Changes at work and employee reactions: Organizational elements, job insecurity, and short-term stress as predictors for employee health and safety. *Scandinavian Journal of Psychology* **2006**, *47*, 541-550, doi:10.1111/j.1467-9450.2006.00548.x.

Størseth, F. Affective job insecurity and risk taking at work. *International Journal of Risk Assessment & Management* **2007**, *7*, 189-204, doi:10.1504/IJRAM.2007.011731.

Tang, T.L.-P.; Singer, M.G.; Roberts, S. Employees' perceived organizational instrumentality: An examination of the gender differences. *Journal of Managerial Psychology* **2000**, *15*, 378-406, doi:10.1108/02683940010337112.

Teng, E.; Zhang, L.; Qiu, Y. Always bad for creativity? An affect-based model of job insecurity and the moderating effects of giving support and receiving support. *Economic and Industrial Democracy* **2018**, 10.1177/0143831X17744026, doi:10.1177/0143831X17744026.

Vander Elst, T.; De Cuyper, N.; Baillien, E.; Niesen, W.; De Witte, H. Perceived control and psychological contract breach as explanations of the relationships between job insecurity, job strain and coping reactions: Towards a theoretical integration. *Stress and Health: Journal of the International Society for the Investigation of Stress* **2016**, *32*, 100-116, doi:http://dx.doi.org/10.1002/smi.2584.

Vander Elst, T.; De Witte, H.; De Cuyper, N. The Job Insecurity Scale: A psychometric evaluation across five European countries. *European Journal of Work and Organizational Psychology* **2014**, *23*, 364-380, doi:10.1080/1359432X.2012.745989.

Wang, H.; Liu, X.; Luo, H.; Ma, B.; Liu, S. Linking procedural justice with employees work outcomes in China: The mediating role of job security. *Social Indicators Research* **2016**, *125*, 77-88, doi:10.1007/s11205-014-0828-y.

Wang, H.C.; Ma, B.; Liu, X.; Liu, S.S. Job security and work outcomes in China: Perceived organizational support as mediator. *Social Behavior and Personality* **2014**, *42*, 1069-1076, doi:10.2224/sbp.2014.42.7.1069.

Wang, H.J.; Lu, C.Q.; Siu, O.L. Job insecurity and job performance: The moderating role of organizational justice and the mediating role of work engagement. *Journal of Applied Psychology* **2015**, *100*, 1249-1258, doi:10.1037/a0038330.

Wang, H.-j.; Lu, C.-q.; Lu, L. Do people with traditional values suffer more from job insecurity? The moderating effects of traditionality. *European Journal of Work and Organizational Psychology* **2014**, *23*, 107-117, doi:10.1080/1359432X.2012.712751.

Wang, S.L.; Zhou, H.M.; Wen, P. Employment modes, charismatic leadership and organizational citizenship behavior: Explanations from perceived job security. *Pakistan Journal of Statistics* **2014**, *30*, 827-836.

Wilson, M.G.; Dejoy, D.M.; Vandenberg, R.J.; Richardson, H.A.; McGrath, A.L. Work characteristics and employee health and well-being: Test of a model of healthy work organization. *Journal of occupational and organizational psychology* **2004**, *77*, 565-588, doi:10.1348/0963179042596522.

Wong, Y.-T. Job security and justice: predicting employees' trust in Chinese international joint ventures. *The International Journal of Human Resource Management* **2012**, *23*, 4129-4144, doi:10.1080/09585192.2012.703423.

Wong, Y.T.; Wong, Y.W. Workplace guanxi and employee commitment to supervisor in Chinese international joint ventures. *Journal of Chinese Human Resources Management* **2013**, *4*, 39-57, doi:10.1108/JCHRM-01-2013-0003.

Wong, Y.-T.; Ngo, H.-Y.; Wong, C.-S. Antecedents and outcomes of employees' trust in Chinese joint ventures. *Asia Pacific Journal of Management* **2003**, *20*, 481-499, doi:10.1023/A:1026391009543.

Wong, Y.-T.; Wong, C.-S.; Ngo, H.-Y.; Lui, H.-K. Different responses to job insecurity of Chinese workers in joint ventures and state-owned enterprises. *Human Relations* **2005**, *58*, 1391-1418, doi:10.1177%2F0018726705060243.

Ye, J.; Cardon, M.S.; Rivera, E. A mutuality perspective of psychological contracts regarding career development and job security. *Journal of Business Research* **2012**, *65*, 294-301, doi:10.1016/j.jbusres.2011.03.006.

Yi, X.; Wang, S.H. Revisiting the curvilinear relation between job insecurity and work withdrawal: The moderating role of achievement orientation and risk aversion. *Human Resource Management* **2015**, *54*, 499-515, doi:10.1002/hrm.21638.

Yousef, D.A. Satisfaction with job security as a predictor of organizational commitment and job performance in a multicultural environment. *International Journal of Manpower* **1998**, *19*, 184-194, doi:10.1108/01437729810216694.

Zoghbi-Manrique-de-Lara, P.; Ting-Ding, J.M.; Guerra-Baez, R. Indispensable, expendable, or irrelevant? Effects of job insecurity on the employee reactions to perceived outsourcing in the hotel industry. *Cornell Hosp. Q.* **2017**, *58*, 69-80, doi:10.1177/1938965516648791.
